# A Review of Commercial Developments and Recent Laboratory Research of Dialyzers and Membranes for Hemodialysis Application

**DOI:** 10.3390/membranes11100767

**Published:** 2021-10-07

**Authors:** Noresah Said, Woei Jye Lau, Yeek-Chia Ho, Soo Kun Lim, Muhammad Nidzhom Zainol Abidin, Ahmad Fauzi Ismail

**Affiliations:** 1 Advanced Membrane Technology Research Centre (AMTEC), School of Chemical and Energy Engineering, Universiti Teknologi Malaysia, Skudai 81310, Malaysia; noresahsaid@yahoo.com (N.S.); nidzhom1992@gmail.com (M.N.Z.A.); afauzi@utm.my (A.F.I.); 2 Centre of Urban Resource Sustainability, Department of Civil and Environmental Engineering, Institute of Self-Sustainable Building, Universiti Teknologi PETRONAS, Seri Iskandar 32610, Malaysia; yeekchia.ho@utp.edu.my; 3 University Malaya Primary Care Research Group (UMPCRG), Department of Primary Care Medicine, Faculty of Medicine, University of Malaya, Kuala Lumpur 50603, Malaysia; limsk@ummc.edu.my

**Keywords:** commercial dialyzer, dialysis, membrane, dialyzer design, hemodialysis, blood

## Abstract

Dialyzers have been commercially used for hemodialysis application since the 1950s, but progress in improving their efficiencies has never stopped over the decades. This article aims to provide an up-to-date review on the commercial developments and recent laboratory research of dialyzers for hemodialysis application and to discuss the technical aspects of dialyzer development, including hollow fiber membrane materials, dialyzer design, sterilization processes and flow simulation. The technical challenges of dialyzers are also highlighted in this review, which discusses the research areas that need to be prioritized to further improve the properties of dialyzers, such as flux, biocompatibility, flow distribution and urea clearance rate. We hope this review article can provide insights to researchers in developing/designing an ideal dialyzer that can bring the best hemodialysis treatment outcomes to kidney disease patients.

## 1. Introduction

One of the major health problems suffered by many people all over the world is renal failure. Over the course of the 21st century, the number of chronic kidney disease patients has increased significantly, where these patients suffer from an incapability of filtering blood by removing waste products from the body. Approximately 10% of the world population is reported to suffer from chronic kidney failure, among which one-quarter are very critical [1]. Currently, dialysis is a lifesaving treatment to approximately 3.4 million people worldwide [2]. A report revealed that China (18%) and the USA (16%) are the top two countries accounting for over one-third of the total number of dialysis patients in the world [3]. They are followed by Japan (10%), India (5%) and Brazil (4%) [3]. It is projected that the global dialysis population will continue to grow yearly and reach close to 5 million by 2025 [4].

In general, patients who suffer from renal failure can choose to undergo either dialysis treatment or kidney transplantation [5]. As the chance to receive kidney transplant is very low, dialysis is required for patients while they wait for a suitable kidney donor. It must be noted that peritoneal dialysis, which uses the lining of the human abdomen (also known as peritoneum) and a cleansing solution (i.e., dialysate) to clean the blood from waste and toxic substances, may not be suitable for every individual, especially those who are obese or have had multiple prior abdominal surgeries [6]. Thus, hemodialysis (also spelled haemodialysis) is considered a highly successful treatment that can provide life support for end-stage kidney disease (ESKD) patients to live. Unlike peritoneal dialysis, hemodialysis uses man-made semipermeable polymeric membrane in an extracorporeal system to filter the blood.

In 1943, Willem Kolff was the first person to successfully treat a patient with hemodialysis using a cellulosic membrane 12 times, although the patient ultimately died because of vascular access failure [7]. Long-term hemodialysis treatment was only first reported in 1960, when Belding and his colleagues at the University of Washington, United States of America (USA), designed shunted cannulas to prevent the destruction of blood vessels, enabling repeated hemodialysis sessions [8]. Fast forward to the present, the semipermeable membrane technology has been commercially used for hemodialysis treatment for patients who suffer from acute kidney disease (also known as acute kidney injury, AKI) and ESKD. The main component of a dialysis machine is the dialyzer, where a semipermeable hollow fiber membrane is situated. The role of the dialyzer is to act similarly to glomerulus to remove excess wastes and fluid from the blood; thus, it is often called an “artificial kidney” [9].

Geographically, the hemodialysis market is segmented into North America, Europe, Asia-Pacific, Latin America, the Middle East and Africa. In 2020, North America accounted for the largest share of global hemodialysis, followed by Europe and the Asia-Pacific [10]. Currently, there are more than 200,000 people in North America that require dialysis treatment based on the statistics released by the USA [11]. In terms of the global market, hemodialysis and peritoneal dialysis are expected to grow at a projected compound annual growth rate of 4.4% in the coming years (between 2020 and 2027) and reach USD 17.69 billion in 2027 [10]. According to Canaud et al. [12], hemodialysis treatment will be substantially more personalized to patients in 2030, with a focus on cardio-protection, volume management, arrythmia surveillance and avoidance of anticoagulation.

Currently, global products and services of hemodialysis are dominated by several companies, namely Fresenius Medical Care AG and Co. KGaA (Bad Homburg, Germany), B. Braun Mesulgen AG (Hessen, Germany), Membrane GmbH (Radeberg, Germany), Da Vita Healthcare Partners, Inc. (Denver, CO, USA), Baxter International, Inc. (Deerfield, IL, USA), WEGO Healthcare Co., Ltd. (Shenzhen, China), Nipro Corporation (Osaka, Japan), Toray Industries, Inc (Tokyo, Japan), Asahi Kasei Medical Co., Ltd. (Tokyo, Japan) and Gambro AB (Lund, Sweden) [13,14,15]. These companies are competing in the same niche to produce a high-quality dialyzer, which is the heart of hemodialysis treatment.

To date, Fresenius’s polysulfone (PSf)-based dialyzer is widely acknowledged as the product that offers optimal biocompatibility, good solute removal and low complement activation [16]. This dialyzer has always been used as the standard in the development of novel dialyzers over the years. Lately, Fresenius launched a new class of PSf-based dialyzers that combine an innovative housing design and an advanced hemodialysis membrane and named it as FX-class dialyzers. Besides PSf, other polymeric materials, such as polyethersulfone (PES) and polymethylmethacrylate (PMMA), are also used for making dialyzers. For instance, Polyflux^®^ and Revaclear^®^ dialyzers manufactured by Baxter are made of membranes composed of PES, polyvinylpyrrolidone (PVP) and polyamide (PA) [13,17]. Meanwhile, the Filtryzer^®^ series from Toray Medical Co. Ltd. (Tokyo, Japan) utilizes the membranes made of PMMA to provide excellent biocompatibility coupled with a well-balanced removal capability of middle and small-molecular toxins. The surface of membranes, which is functionalized with vitamin E, is reported to be clinically meaningful in circulating reactive oxygen species that are generated at the membrane–blood interface [17,18]. In the last few years, biomedical key players, such as Nipro (Osaka, Japan) and WEGO (Shenzen, China), are also involved in manufacturing dialyzers. Table 1 summarizes the main dialyzers manufactured by global players and the polymeric materials and sterilization methods used to produce the dialyzers.

Over the years, the development of dialyzers has been focused on the performance of membranes and their biocompatibility and hemocompatibility [19]. Previous relevant review articles were focused on the progress of materials used for the fabrication of dialysis membranes [5,9,20]. More specifically, attention has been paid to addressing the biocompatibility issues of the dialysis membranes [21]. For example, a review written by Irfan et al. [22] discussed the importance of interactions between PES-based membranes and blood elements by emphasizing the activation of blood cells, the adherence of platelets and thrombosis reactions. Salimi et al. [23], on the other hand, reviewed various strategies that could be adopted to enhance the hemocompatibility of polymeric membranes through membrane surface modification/functionalization. Another review written by Ronco and Clark [24] provided an update of commercial dialysis membranes and dialyzers with respect to performance in clinical settings, transport mechanisms and the interaction between the membrane and blood. In 2020, Himmelfarb et al. [8] published a review on the basic epidemiology of kidney failure treated with long-term dialysis and discussed the key epidemiological challenges of the future.

**Figure 1:**
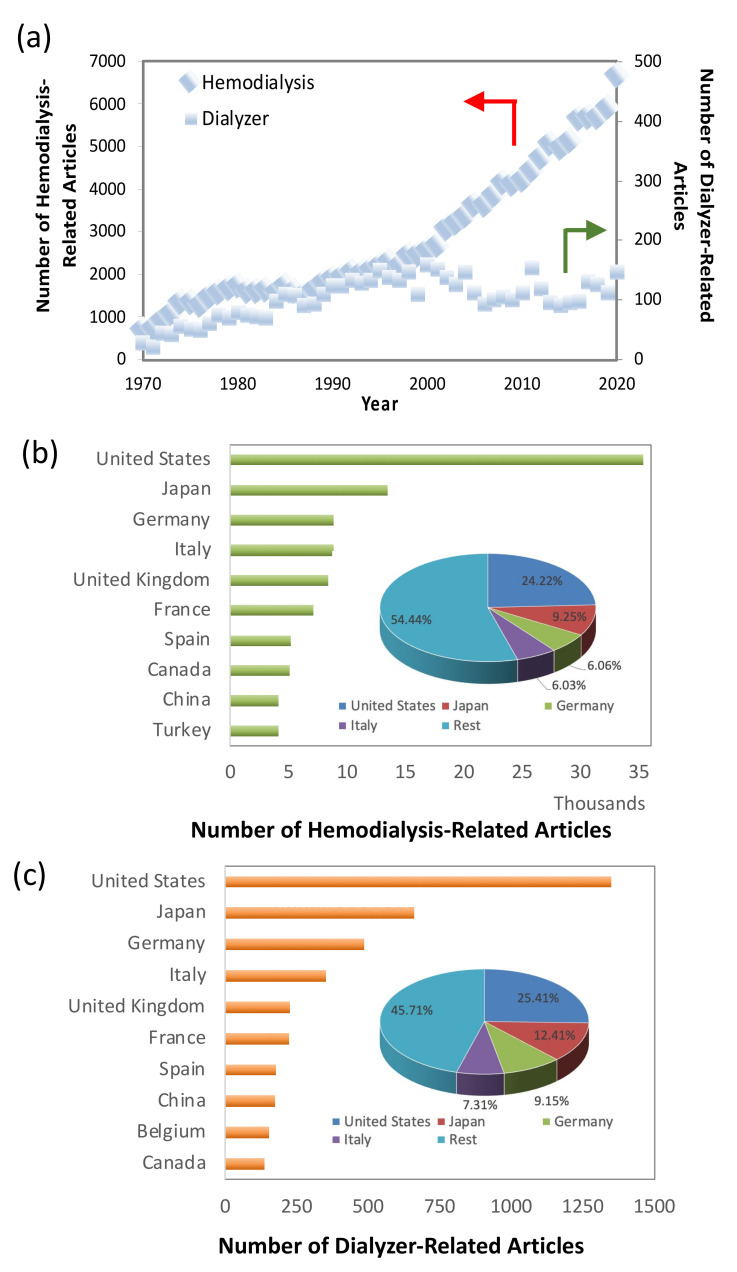
(**a**) Number of research publications related to hemodialysis and dialyzers for the period of 1970–2020 (data from Scopus; assessed on 7 July 2021; search within: article title, abstract, keywords; search documents: “hemodialysis” or “haemodialysis” (for hemodialysis) and “dialyzer" or “dialyser” (for dialyzer)) and (**b**,**c**) number of research papers contributed by top 10 countries for hemodialysis- and dialyzer-related articles (inset: picture chart of country’s contribution in %).

In terms of research activities, Figure 1a shows the number of publications over the past 50 years on the topics of hemodialysis and dialyzers. The significant increase in the publication of articles related to hemodialysis in the last two decades strongly indicates the research interest among scientists worldwide. Figure 1b,c show that the USA, Japan, Germany, Italy and the United Kingdom (UK) are the top five nations in the world producing hemodialysis- and dialyzer-related articles, respectively.

Although the performance of a dialyzer is mainly governed by the dialysis membrane, other factors, such as potting material, blood/dialysate port design and sterilization techniques, also play important roles in producing a promising dialyzer. Efforts should be made to improve the design of dialyzers, including the dialysate and arterial (blood) port, the bundle configuration and the materials used for the construction of the whole device. For example, the blood compartment of an ideal dialyzer should exhibit a low priming volume but maximal blood–membrane contact surface. Meanwhile, the blood ports, which were previously thought as the arterial and venous ends of the unit, are now receiving greater attention due to their importance in optimizing blood flow distribution [25].

The ideal dialyzer should not only exhibit good efficiency and consistent performance for solute removal but also possess a high degree of safety [5,26]. Furthermore, it should be effective in removing all types of uremic toxins while retaining vitamins and other essential proteins, e.g., albumin. In view of this, factors such as the type of dialyzer (vary in terms of membrane formulation, housing selection and sterilization technique), the performance of dialysis membranes and the design of the dialyzer play crucial roles. Currently, there is a knowledge gap related to the development of innovative dialyzers in the laboratory setting because such review articles are outdated and scarce.

Thus, this article aims to provide an up-to-date review on the recent laboratory research of dialyzers for hemodialysis application and to discuss the technical aspects of dialyzer development, which include, but are not limited to, dialysis membrane and dialyzer design. Commercial developments of dialyzers are also reviewed and highlighted in this article. Lastly, the technical challenges in developing dialyzers are also discussed.

## 2. Types of Dialyzers

In general, commercial dialyzers are classified into five categories in accordance with the clearance of β_2_-microglobulin (β_2_-M) and the level of albumin loss (in gram) per 4 h treatment [27]. Table 2 shows the detailed classification of each commercial dialyzer. This classification acts as a guidance for doctors to choose a suitable type of dialyzer for patients based on their health conditions.

Conventionally, dialysis membranes are categorized based on the permeability and sieving coefficient. Permeability is a measure of the clearance rate of molecules of middle molecular weight or sometimes defined using β_2_-M. Dialyzers with an ultrafiltration coefficient (*K_UF_*) of <10 milliliters per hour per mmHg transmembrane pressure (mL/h/mmHg) and >20 mL/h/mmHg under usual clinical flow and ultrafiltration conditions are typically defined as low-flux and high-flux membranes, respectively. The coefficient is determined from in vitro tests and corresponds to water permeability.

Maintaining the level of albumin in blood is essential for all dialysis membranes, as excessive albumin loss could trigger albumin loss syndrome (hypoalbuminemia), which could result in morbidity for the patients and even death [30,31]. From Table 2, albumin loss tends to increase with increasing *K_UF_* due to the enlargement of membrane pore sizes, particularly for super high-flux dialyzers. The rate of mass transfer for large solutes, such as albumin, is also greatly dependent on *K_UF_* (convective transport) rather than diffusive transport [32].

Compared to the high-flux dialyzer, super high-flux dialyzers possess an enhanced convection or adsorption rate. This type of dialyzer is mainly used to treat septic patients with AKI and provides the control of uremia and fluid status together with cytokine removal [33]. In addition, super high-flux dialyzers are found to be more effective in reducing plasma homocysteine [34]. However, a critical drawback of using this kind of dialyzer is the significant losses of albumin protein, hormones and other essential vitamins for patients.

Medium cut-off (MCO) dialyzers are typically used to remove larger middle molecules that are associated with symptoms of uremic toxin accumulation in blood. The membrane of MCO dialyzers is characterized to exhibit a relatively higher number of pores with an extremely narrow pore size distribution [27]. Such properties provide the dialyzer with a higher permeability, better convective transport and an improved removal rate of middle molecules [35]. Nevertheless, one major concern of using this type of dialyzer is the risk of albumin loss through the more permeable membrane structure. In the study conducted by Kirsch et al. [36], it was reported that MCO dialyzers tended to remove more albumin from blood compared to high-flux dialyzers.

Compared to the abovementioned dialyzers, the protein-leaking dialyzer is rather new [27,29] and still not a commonly used term for clinicians. This dialyzer generally provides a greater clearance against low-molecular-weight proteins and small protein-bound solutes. Clinical trials found that protein-leaking dialyzers tend to improve anemia correction, decrease plasma total homocysteine concentrations and reduce plasma concentrations of glycosylated and oxidized proteins [37]. However, the routine use of protein-leaking dialyzers is still not clear [29]. In addition, specific uremic toxins removed by protein-leaking dialyzers are yet to be fully disclosed. It is also unclear if protein-leaking dialyzers could outweigh the conventional dialyzers used in convective therapies, such as hemofiltration and hemodiafiltration. The amount of protein loss that can be tolerated by a hemodialysis patient in long-term therapy also remained largely unclear. Although some studies reported that protein-leaking dialyzers offer a new solution in improving outcomes of hemodialysis [29], more clinical trials are still required to understand if their benefits could really outweigh their disadvantages.

A high-performance dialyzer should be able to achieve a good clearance of waste and harmful products while minimizing the huge loss of essential substances, such as proteins. A study was previously conducted to better understand if the use of high-flux dialyzers (compared with low-flux dialyzers) could have measurable effects on the survival rate of hemodialysis patients [38]. The findings showed that besides exhibiting higher permeability than that of the low-flux dialyzers, the clearance of high-flux dialyzers against uremic toxins with medium and high molecular weights (e.g., β_2_-M and phosphorus) was also reported to be higher. Furthermore, high-flux dialyzers absorb toxins associated with uremia and tend to reduce cytokines and complement activators, diminishing inflammatory responses. Nevertheless, a separate study reported that there were no significant differences between high-flux and low-flux dialyzers in terms of low-molecular-weight-toxin removal [39]. Practically, low-flux dialyzers are still commonly used in dialysis centers, and one of the main reasons to use low-flux dialyzers is their lower price in comparison to high-flux dialyzers. This has made them more affordable for patients with ESKD.

### 2.1. Low-Flux Dialyzers

Utilizing low-flux dialyzers is a reliable option for acute and chronic dialysis, where a lower rate of fluid removal or *K_UF_* is desired. Low-flux dialyzers allow patients to achieve desired adequacy dialysis goals while offering biocompatibility very similar to that of high-flux dialyzers. Furthermore, low-flux dialyzers are more suitable for pediatric patients that require low dialysate flow rates.

The main target of using low-flux dialyzers is to achieve an efficient low-molecular-weight solute clearance. The small solute removal in low-flux dialyzers is usually obtained primarily by diffusion. Diffusion can be influenced by many factors, such as dialysate/blood flow rates, operating temperature and the surface area of the dialyzer. Assuming all other factors are constant, the diffusion rate during the dialysis process is mainly governed by the concentration gradient between the dialysate and the blood, the dialysate/blood flow rates and the flow distribution (in counter current mode) in their relative compartments. Any possible mismatch between the dialysate and flow distributions can result in a reduction in the diffusion efficiency [40].

**Figure 2:**
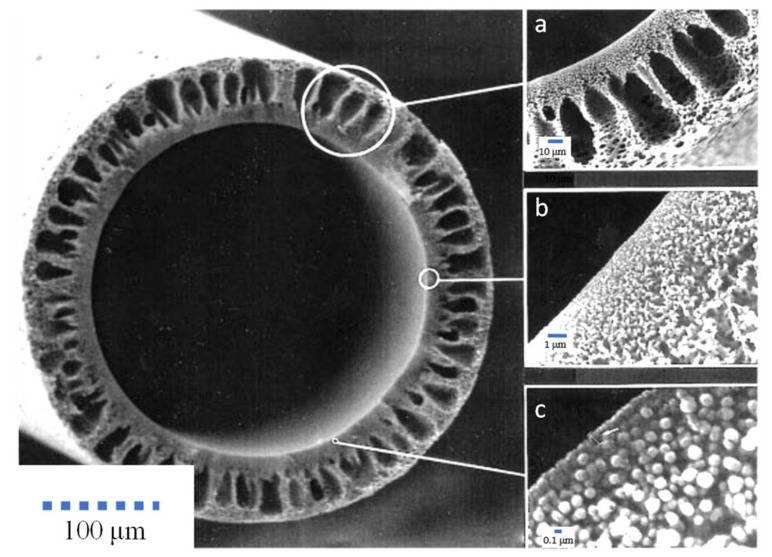
The SEM images of Polyflux^®^ L dialyzer membrane, (**a**) membrane wall showing porous finger-like structure, (**b**) compact sponge-like layer and (**c**) thin dense layer [41].

Among the low-flux dialyzers available on the market, Polyflux^®^ L, Baxter (deerfield, IL, USA) and Hemoflow^™^, Fresenius (Bad Homburg, Germany), are mostly used for low-flux hemodialysis treatment. The Polyflux^®^ L dialyzer consists of a membrane made up of polyarylethersulfone (PAES)/PVP and PA. This hydrophobic membrane can achieve increased endotoxin retention and improved biocompatibility with blood components. In addition, the use of PAES provides the membrane with good mechanical strength and resistance to heat sterilization, while the presence of PVP enhances the membrane diffusive permeability. Figure 2 shows the morphological structure of the hollow fiber membrane used in the Polyflux^®^ L dialyzer. The membrane has a well-defined porous finger-like structure (Figure 2a) for good mechanical support, a compact sponge-like inner structure (Figure 2b) and a dense, blood-contacting skin layer (Figure 2c), which play different roles during hemodialysis treatment.

Hollow fiber membranes have been used as artificial kidneys since the 1960s in order to offer improved geometry with respect to solute mass transfer and blood rheology. It has unique advantages, such as a high surface area/volume ratio (in the blood compartment) and low end-to-end pressure drops. All these features make it as the main choice of configuration in clinical dialysis.

In terms of performance, low-flux dialyzers are still efficient for the removal of low-molecular-weight uremic toxins and are suitable for AKI patients who need temporary dialysis support. Ponikvar et al. [42] compared the performance of a low-flux dialyzer with a high-flux dialyzer for AKI patients and found that there was no significant influence on the survival rate of patients based on the recovery of renal function. Furthermore, low-flux dialyzers offer little or no benefit for the removal of middle molecules and/or large-molecular-weight toxins. The long-term side effects of the accumulation of middle molecules are usually linked to amyloidosis and cardiovascular disease [43].

### 2.2. High-Flux Dialyzers

Hemodialysis treatment using a high-flux dialyzer makes use of highly permeable membranes and accurate fluid flow control to clear the accumulated toxins and water from the blood of patients with ESKD [41]. The criteria of high-flux dialyzers include the effective clearance of target solutes at a faster rate and excellent biocompatibility.

The biocompatibility of high-flux dialyzers is reflected by several parameters, including lower activated serum complement levels, lower reduction in white cell counts, lower oxygen radical production and reduction in interleukin release [44]. The Japanese Society of Dialysis Therapy (JSDT) recommends that the pore size in high-flux dialyzers should be large enough to allow only slight losses of albumin, at a rate of less than 3 g/session in hemodialysis conditions with the blood and dialysate flow rate kept at 200 and 500 mL/min, respectively.

The FX series and Hemoflow^™^ from Fresenius are among the best high-flux dialyzers on the market in terms of separation performance and biocompatibility. Both types of high-flux dialyzers can eliminate low/middle-molecular-weight uremic toxins and achieve the desired sieving coefficient. In terms of performance, the high-flux PSf membrane has a larger surface area with a significantly larger number of fibers, and such dialyzers have the capability to handle higher blood flow rates [14]. Consequently, to maintain the removal of solutes, a high dialysate flow rate is required. However, the implication of exposure to a high volume of dialysate is endotoxin contamination, which infiltrates into the patient’s blood stream. Nevertheless, the PSf membrane can adsorb endotoxin molecules and prevent the endotoxin molecules from entering the patient’s blood. This is due to its hydrophobic–hydrophobic interaction with lipopolysaccharides [45].

**Figure 3:**
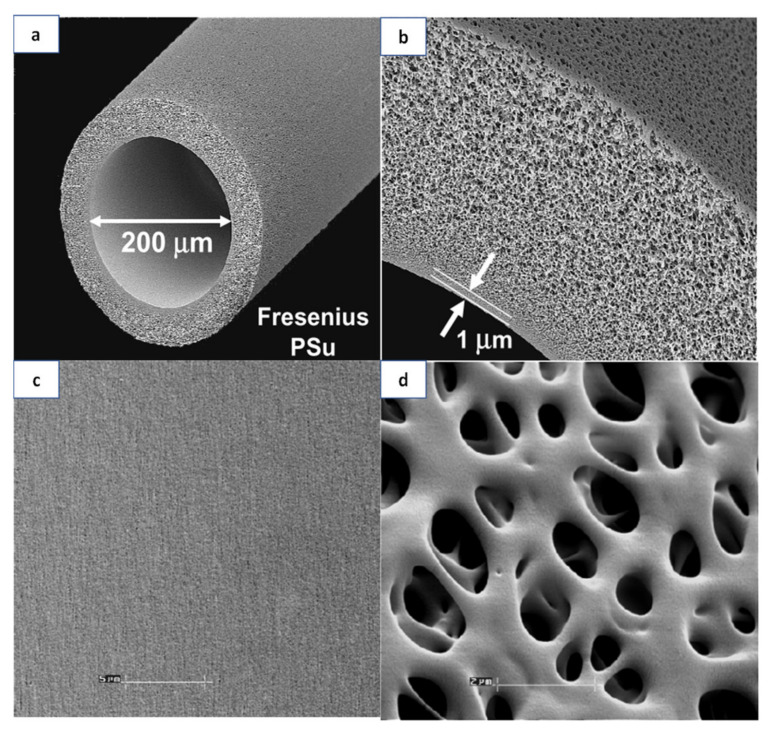
The SEM images of Fresenius Helixone^®^ dialyzer made up of PSf membrane, (**a**) entire membrane structure, (**b**) partial cross-section showing skin layer of about 1 μm, (**c**) inner surface morphology (scale: 5 μm) and (**d**) outer surface morphology (scale: 2 μm) [26].

Figure 3 shows the SEM images of Fresenius’s hollow fiber membrane made of PSf. The membrane structure is significantly different compared to the Polyflux^®^ L dialyzer membrane as shown Figure 2. This membrane comprises a very thin internal skin layer (1 μm) surrounded by a thick sponge-like layer. Other important features of this membrane are its smooth pore texture for increased blood compatibility, homogenous porous structure and ideal pore size for the perfect retention of albumin. It must be pointed out that the structural morphology of the commercially available synthetic hollow fiber dialysis membranes can be very much different with varying degrees of physical structure, ranging from minimum asymmetry (sponge like) to maximum asymmetry (finger like) [24].

The new generation of high-flux dialyzers, such as Polyflux^®^ H and Revaclear^®^ dialyzers, offers several important advantages to patients. These dialyzers composed of a unique synthetic membrane made of PAES, PVP and PA can minimize solute diffusion resistance, allowing the efficient removal of uremic toxins. Meanwhile, the smaller inner diameter of the hollow fiber (~190 μm) can promote its contact with the blood owing to the existence of turbulent flow. In particular, the Polyflux^®^ dialyzer shows an efficient removal of urea, superior β_2_-M clearance and lower thrombogenicity resulting from excellent membrane transport properties, uniform pore shape and distribution, minimum tortuosity and higher porosity [46].

From our point of view, although high-flux dialyzers do not achieve the solute removal profile of a glomerular filtration barrier, at present, they can still be a good option for patients. However, there is concern that the easier passage of water through a high-flux dialyzer could make it prone to water borne contaminants, such as endotoxins (fragments of bacteria) [47].

## 3. Overview of Hemodialysis Performance Evaluation and Standard

The performance evaluation of hemodialysis treatment is very much based on the technical properties and capabilities of the dialyzer. In specific, hemodialysis performance is determined by the dialyzer’s solute clearance and ultrafiltration characteristics [48]. Solute clearance generally corresponds to the removal rate of solutes, such as urea, creatinine, phosphate, inulin vitamin B_12_ and β_2_-M, and is governed by the membrane properties, such as pore size, wall thickness and surface area. Of all solutes, the removal rate of urea is the point of reference since it is used conventionally in the hemodialysis dose calculation to measure small-molecule clearance. An accurate prediction of dialyzer urea clearance during dialysis is essential when prescribing therapy using urea kinetic modeling [49]. According to the Hemodialysis Quality and Standards set by the Ministry of Health Malaysia [50], the quality of hemodialysis treatment must achieve a urea reduction rate of more than 65% for at least 90% of patients.

Solute removal in hemodialysis occurs through a combination of diffusion, convection and adsorption. The uremic solutes removed by hemodialysis are generally divided into four main categories, which are (a) small water-soluble compounds (e.g., urea) with size <500 Dalton (Da), (b) a middle molecular weight of 500–15,000 Da, (c) large molecules with size >15,000 Da and (d) protein-bound molecules (e.g., homocysteine of 135 Da and p-cresol of 108 Da). However, when describing the in vitro performance of dialyzers, many studies only considered the small solute and middle-molecule results. Table 3 compares the differences between small solutes and middle-molecule toxins.

### 3.1. Dialyzer Urea Clearance

Before prescribing the treatment dose, the dialyzer urea clearance during hemodialysis should be known. Vilar and Farrington [60] reported that the concentration of urea and creatinine measured can somehow be misleading. Currently, the prescription of the treatment is carried out in relation to the urea clearance using the urea reduction rate (URR) or by the urea kinetic model as expressed in Equation (1).

(1) $$\mathrm{Dialysis}\;\mathrm{adequacy}\;=\frac{K\times t}{V}$$

where *K* is the dialyzer urea clearance (mL/min), *t* is the duration of dialysis (min), and *V* is the volume of urea distribution (mL).

As for the urea kinetic model, a lower dose (Kt/V < 1.0) can cause short-term mortality for patients. Hence, the recommended standard for Kt/V is set at >1.2. For the removal of other solutes, such as creatinine, a clearance of 70–95% is required. Other than the properties of the dialyzer itself, solute clearance also depends on the blood and dialysate flow conditions during the operation. For the optimization of toxin clearance, it is very important to maximize the efficiency of the dialyzer by increasing dialysate flow rate (*Q_d_*) and blood flow rate (*Q_b_*) as tolerated by patients.

### 3.2. Dialysate Fluid

The dialysate (also known as dialysis fluid) is a solution containing water, electrolytes and salts. In the blood of hemodialysis patients, there is a high concentration of waste [48]. Dialysate with a low concentration is able to draw toxins from the blood due to the concentration difference. Waste moves through the dialyzer to create an equal amount on both sides. The electrolytes are required in the dialysis solution in order to balance the electrocytes in the patient’s blood [61]. Table 4 shows the standard dialysate composition and the respective concentration range [48]. Besides the common composition of the dialysate, there are few studies related to the usage of acetate and citrate dialysate [62,63]. Thousands of patients in the USA nowadays have been treated with hemodialysis fluid based on citric instead of acetic acid. Gabutti et al. [62] compared the impacts of citrate- and acetate-based dialysate on dialysis patients and reported that the citrate dialysate offered positive impact on the dialysis efficiency and acid-base status, which significantly contributed to an improved hemodialysis process.

Basically, low-molecular pathogenic substances are removed by diffusion during dialysis. The flow conditions of blood and dialysis fluid in the dialyzer have significant influence on the dialysis efficiency. In the event of channeling or stagnation occurring in the flow channels of the blood or dialysis fluid, the differences in the concentrations could deteriorate the dialysis efficiency. Thus, maintaining a uniform flow for both fluids is important to achieve high dialysis efficiency.

### 3.3. Flux and Efficiency of Dialyzer

Over the years, there has been a high demand for dialyzers with higher efficiency and better flux. Flux refers to the ability to filter plasma, while efficiency refers to solute clearance at a given *Q_d_* and *Q_b_*. A dialyzer with a membrane flux of less than 10 mL/h/mmHg is categorized as a low-flux dialyzer, while a dialyzer with a flux of >20 mL/h/mmHg is considered a high-flux dialyzer [63]. The main advantage of using a high-flux dialyzer is its high removal rate against middle-molecular uremic toxins [60].

Meanwhile, dialyzer efficiency with respect to the solute clearance against urea is normally measured at a *Q_d_* of 500 mL/min and *Q_b_* of 200 mL/min to maximize the diffusion [50]. The transport of urea can be easily evaluated as long as the solutes (80–100 mg/mL for urea) are maintained at high levels (80–100 mg/mL for urea) in the reservoir of the blood compartment. However, a study reported that dialyzer efficiency could be more accurately measured in terms of the mass transfer coefficient, *K_o_A* [48].

*K_o_A* is a measure of dialyzer efficiency in removing urea or other solutes of similar molecular weight. It is the maximum theoretical clearance of the dialyzer in mL/min for a given solute at infinite blood and dialysate flow rates. For any given membrane, *K_o_A* is proportional to the surface area of the membrane in the dialyzer, although there is a drop-off in the gain in *K_o_A* as the membrane surface area becomes very large. Dialyzers with *K_o_A* values less than 500 mL/min are usually used for small patients, while *K_o_A* values of 500–700 mL/min represent moderate efficiency for high-flux dialyzers. For patients with a large body size, dialyzers with *K_o_A* values greater than 700 mL/min are used in order to achieve high-efficiency dialysis for 4 h treatment sessions [40,64].

Since the pore sizes of a membrane are variably distributed and the number of larger pores is usually fewer than the number of smaller pores, relatively small solutes, such as urea, can easily transport through these pores regardless of their size. The larger molecules, such as vitamin B_12_ and β_2_-M, however, can only pass through the larger pores. Because of this, the clearance of larger solutes is mainly governed by the membrane and is less dependent on the *Q_b_* and *Q_d_* [49]. However, for the optimization of toxin clearance, it is very important to improve the efficiency of the dialysis membrane by manipulating *Q_b_* and *Q_d_*. There are several criteria that can be used to evaluate the performance of dialysis. These include dialyzer efficiency, the efficiency of uremic solute removal and biocompatibility. Some of these parameters are assessed by the removal rates of a uremic solute and other biochemical markers [48,65].

The removal rate or clearance of a uremic toxin is calculated with respect to *Q_b_*. When tested in a real environment, the urea clearance increases steadily as a function of *Q_b_* in the range of 200–450 mL/min [48]. However, it must be pointed out that a dialyzer might not be able to transport urea at the same efficiency when very high flow rates are employed. If this happens, the urea concentration at the outlet of the dialyzer would increase accordingly. In other words, the percentage of the urea inflow into the dialyzer decreases.

Cheung and Leypoldt [66] previously reported that the *K_o_A* of a dialyzer will increase with increasing blood and dialysate flow rates. This is because a decrease in the thickness of a stagnant fluid layer could reduce the resistance for urea to transfer. Noda et al. [67] found that the dialysate flow rate had a greater impact on enhancing the *K_o_A* compared to the blood flow rate because a high concentration gradient is maintained during hemodialysis in order to improve the flow distribution in the dialysate compartment. This effect was more obvious when the *Q_d_* value was set as at least twice the value of *Q_b_*. This finding was in line with the work of Vilar and Farrington [60], in which the researchers reported that the solute removal efficiency is proportional to the flow rate of the dialysate.

## 4. Recent Development of Dialyzers

### 4.1. Polymeric Materials for Membranes

A literature search revealed that most of the hemodialysis membranes found in the current market are made of several main types of polymeric materials. In the early stage of dialysis membrane development, Cuprophan^®^ prepared by the cuprammonium process was one of the most commonly used membranes [68]. This membrane was fabricated by dissolving cellulose in an ammonia/copper solution followed by precipitation in acid. Although it was cheap to produce, it created adverse biological reactions in patients, such as leucopenia and the inhibition of granulocyte metabolism.

Table 5 compares the different types of polymers used for fabricating hemodialysis membranes. In brief, the choice of polymers for commercial hemodialysis membranes has remained similar for the past 30 years, although many new types of polymeric materials have been developed and tested in the laboratory setting. Some of the most used polymeric materials for commercial hemodialysis membranes are PSf, PES, PA and EVAL.

Among these polymers, PSf and PES are the most popular materials used in hemodialysis application. Fast forward to the present, PSf- and PES-based membranes are still relevant in the market and always show better performance and biocompatibility compared to other synthetic polymers. Besides exhibiting outstanding oxidative, thermal and hydrolytic stability, both PSf- and PES-based membranes can also endure many kinds of sterilized processes. Furthermore, these membranes offer high permeability for low- and middle-molecular-weight proteins while retaining higher proteins during hemodialysis. However, when in contact with blood, the proteins tend to rapidly adsorb onto the membrane surface, forming a protein layer that may lead to undesirable results, such as an inadequate compatibility. Because of this issue, an injection of anti-coagulants is needed during clinical application.

The internal skin layer of the hollow fiber membrane is of importance because it acts as the barrier for solute separation and is the membrane portion that contacts directly with the blood. There were studies related to the modification of PSf and PES using a sulfonation or carboxylation technique to improve the materials’ hydrophilicity prior to its use for membrane fabrication [74,75,76,77]. Besides improving hydrophilicity, the sulfonated PSf and PES membranes are found to exhibit an increased negative charge that is important in inducing electrostatic repulsion with negatively charged proteins. Meanwhile, carboxylic groups could be introduced into the backbone of the polymer in a similar way of sulfonation (aromatic substitution reaction), in which a carboxylic group replaces a hydrogen atom at the aromatic ring of the polymer.

Wang et al. [77] successfully sulfonated the PES using sulfuric acid and chlorosulfonic acid as sulfonation agents. The resultant sulfonated PES (SPES) was then blended with PES in the concentration of 5–50% to form a membrane. The SPES-modified membrane was reported to effectively minimize fouling caused by bovine serum albumin (BSA), reducing its adsorption rate from 30 to 15 μg/cm^2^ and prolonging its blood coagulation time. Improved blood compatibility was also reported by Zhang et al. [78] when 4% of SPES was introduced into a membrane made of 16% PES. The results indicated that the morphology of the SPES-modified membranes was altered, which led to a greater performance. The APTT, plasma recalcification time (PRT) and platelet adhesion test also showed that the anticoagulant activity of the SPES-modified membrane had been greatly improved, suggesting its greater blood compatibility. PRT is normally used to monitor the time taken for blood to clot and to determine the deficiency of the factor responsible for blood clotting [78].

In addition to PSf and PES, poly(lactic acid) (PLA) is also commonly used by researchers for the synthesis of hemodialysis membranes. The applications of PLA are typically found in food packaging, tissue engineering scaffolds and bone fixation. Due to its biocompatibility characteristics, PLA has a great potential to replace petrochemical-based polymers. Goa et al. [79] utilized PLA as the main membrane-forming material to develop a dialysis membrane. After simulating dialysis for 4 h, the urea and lysozyme clearances of the PLA membrane were reported at 74.6 and 13.7%, respectively. Meanwhile, the membrane BSA rejection was recorded at 90.8%. Besides exhibiting better platelet adhesion, the PLA membrane required a shorter PRT than that of PSf membranes. Nevertheless, it must be pointed out that PLA is hydrophobic in nature and, thus, has a high tendency to foul when exposed to blood protein. In view of this, further modification is needed to solve its fouling issue.

In order to improve the pore structure and dialysis performance of a PLA membrane, Yu et al. [80] incorporated polysulfone-graft-poly(lactic acid) (PSF-EDA-26) into the PLA membrane matrix. The membrane water flux declined with the increasing content of PSf-EDA-26. When 5 wt% of PSf-EDA was incorporated, the resultant membrane barely produced any flux at an operating pressure of 1 bar. With respect to solute clearance and retention, by increasing the PSf-EDA-26 content, the clearances of urea and lysozyme were found to decrease, while BSA retention was increased. The reduced membrane permeability coupled with the increased BSA retention could be attributed to the formation of a smaller surface pore size (denser skin layer). Of the membranes fabricated, the PLA membrane modified by 3 wt% PSf-EDA-26 was found to be suitable for hemodialysis processes as it displayed a reasonably high water flux with a high BSA retention (95%). In addition, it showed 65 and 18% clearance against urea and lysozyme, respectively.

Surface modification of the PLA-based membrane was also carried out by Zhu et al. [81] by blending the PLA membrane with poly(lactic acid)-block-poly(2-hydroxyethyl methacrylate) (PLA-PHEMA) copolymers in an attempt to enhance the antifouling and hydrophilicity of PLA materials. The findings of this work clearly showed the potential of PLA-HEMA as an effective agent for optimizing the characteristics of PLA membranes for hemodialysis application. Upon the incorporation of 15 wt% block copolymers, the modified membrane demonstrated an improved antifouling property (i.e., higher flux recovery rate) and hemocompatibility (i.e., suppressed platelet adhesion and prolonged plasma recalcification) because of the improved surface hydrophilicity. Furthermore, the modified membrane showed excellent creatinine and urea clearances (>0.70 mL/min) when tested using a mimic blood composed of urea (1.5 g/L), creatinine (0.2 g/L) and lysozyme (0.2 g/L) in saline and distilled water as the dialysate.

**Figure 4:**
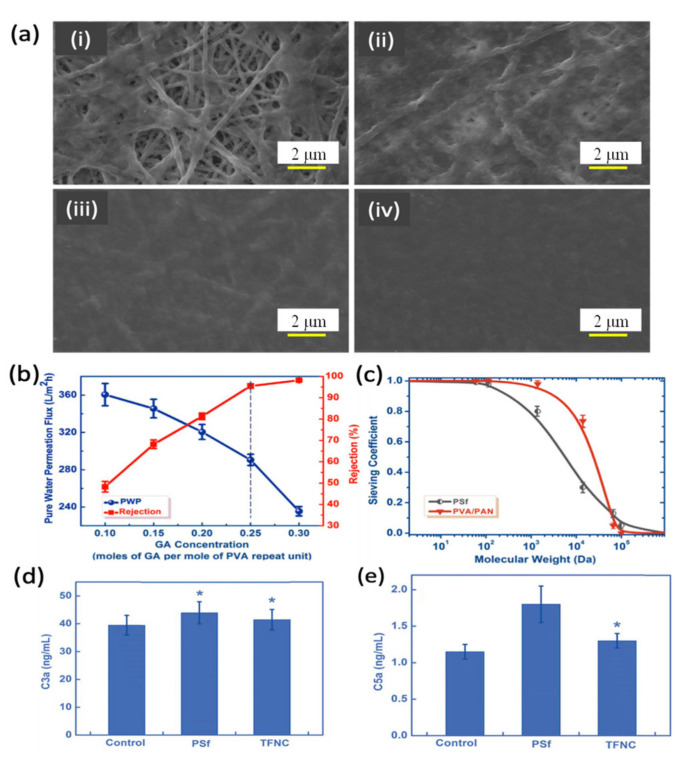
(**a**) SEM images of nanofibrous-based TFNC membranes with different concentrations of PVA coating solution, (**i**) 1 wt%, (**ii**) 1.5 wt%, (**iii**) 2.0 wt% and (**iv**) 2.5 wt%. (**b**) Relationship between pure water flux and BSA rejection of the nanofibrous-based TFNC membranes with the degree of cross-linking of the PVA hydrogel coating. (**c**) Sieving coefficients of best TFNC (i.e., PVA/PAN) membrane with conventional benchmark PSf membrane against several important markers. (**d**,**e**) Concentration of anaphylatoxins C3a and C5a for the samples with whole blood [82].

Thin-film nanocomposite (TFNC) membranes were also studied for hemodialysis application. Yu et al. [82] developed a TFNC membrane composed of a two-tier composite structure, namely a thin hydrophilic layer of chemically cross-linked PVA atop an electrospun PAN nanofibrous layer in an attempt to remove middle-molecule toxins while retaining albumin. Figure 4a compares the surface morphology of the membranes coated with PVA solutions of varying concentration. As shown, when the concentration of the PVA coating solution was lower than 2 wt%, the PVA selective skin layer did not perfectly form on the PAN nanofibrous layer (Figure 4a i and ii). This could be due to the low viscosity of the low-concentrated PVA solutions. By increasing the PVA concentration from 1.5 to 2.0 and 2.5 wt% (Figure 4a iii and iv), the surface of the PAN nanofibrous layer was perfectly covered by a thin selective layer made of PVA. Figure 4b shows the impact of glutaraldehyde (GA) as a cross-linking agent on the water flux and rejection of the TFNC membrane with a 2 wt% PVA coating. It was found that a high water flux (290 L/m^2^·h) with a high BSA rejection (95%) could be achieved when the GA/PVA repeat unit ratio was 0.25. These findings were due to the unique membrane structure that formed, which offered good mechanical strength and comparable hemocompatibility. The sieving curve (SC) of the membrane as shown in Figure 4c clearly indicates that the PVA/PAN TFNC membrane has a sharper separation curve compared to the conventional PSf membrane, indicating its great selectivity, which is more suitable for hemodialysis. Owing to the uniform pore size distribution, the developed TFNC membrane demonstrated a high SC for urea (1.0) and lysozyme (0.75) but a low SC value for BSA (0.05). In Figure 4d, both membranes show no significant difference from the control sample in C3a levels. However, for the concentration of C5a (Figure 4e), only the PSf membrane showed a slightly higher concentration than that of the control sample. The presented results of complement activation indicated that the TFNC membrane showed comparable properties to those of the conventional PSf membrane.

**Figure 5:**
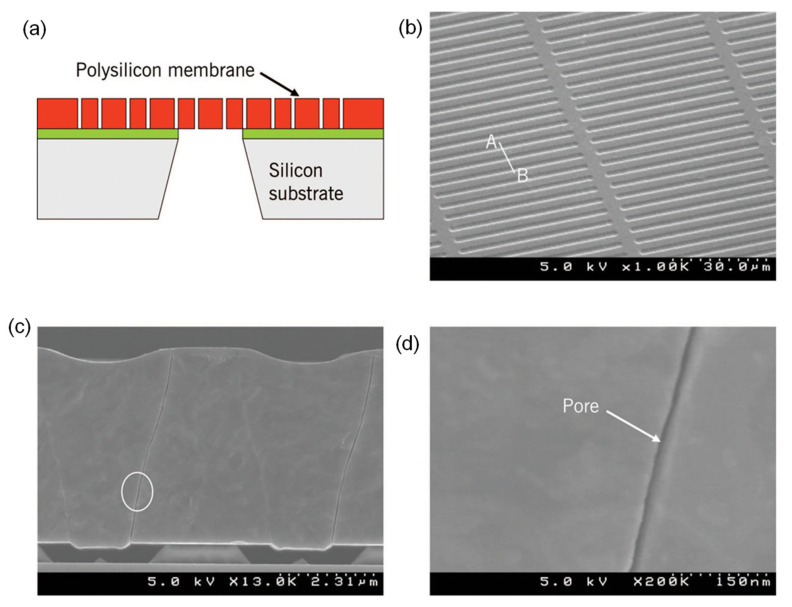
(**a**) Illustration of silicone nanopore membrane (SNM) fabricated using microelectromechanical system (MEMS) technology and SEM images of SNM, (**b**) image of membrane showing uniformly spaced array of slit pores, (**c**) image of the non-tortuous path of the pore and (**d**) close-up image of slit pore showing the smooth surface characteristic [85].

Kim et al. [83] developed a flat sheet silicone-based membrane (SNM) for portable hemodialysis application using the microelectromechanical fabrication technique. Figure 5 shows an illustration SNM and the SEM images of SNM at different views. The SNM was designed to mimic the slit pore geometry of the kidney glomerular in order to achieve an order-of-magnitude higher permeability over commercial available hollow fiber membranes [84]. The SNM has a biomimetic slit pore geometry and a uniform pore size distribution that allow for solute selectivity and permeability. A schematic of the SNM at two support structure thicknesses demonstrating the change in diffusion length was produced. The standard SNM with a 400-μm-thick support structure was estimated to have a longer diffusion path and decreased diffusive transport, while the diffusive SNM with a reduced support structure (100 μm) was estimated to have improved diffusive clearance owing to the decreased resistance from the support structure. A CFD analysis was also conducted to examine whether the backside cavity created by the 100 μm support would lead to stagnant flow under the membrane. The flow was quickly fully developed within the cavity even at a low flow rate. At a flow rate as low as 1 mL/min, there were only minimal regions of stagnant flow under the membrane. Additionally, the authors reported that the diffusive SNM with thinner support structures could achieve over twofold improved clearances in comparison to the standard SNM with superior relative β_2_-m clearance compared to high-flux dialyzers.

### 4.2. Incorporation of Additives

#### 4.2.1. Organic Materials

The surface wettability of hydrophobic membranes could be easily improved by blending them with hydrophilic materials, such as polyvinylpyrrolidone (PVP) [86,87] and polyethylene glycol (PEG) [88,89]. PVP is highly polar, water soluble, amphiphilic, nonionic and physiologically inert. It is available in various molecular weights, either in liquid or powder form. Chakrabarty et al. [90] investigated the effects of different types of PVP (24, 40 and 360 kDa) on the morphology and permeation of PSf membranes, and the findings showed that PVP with a larger molecular weight tended to form a denser membrane structure with less macrovoids and a smoother surface. Owing to the hydrophilic nature of PVP, the modified membrane was reported to exhibit an improved antifouling property and blood compatibility [91]. It must also be noted that PVP could serve as the common pore former during the phase inversion process, which leads to an increase in membrane porosity [92].

Using PEG as an additive, Chakrabarty et al. [90] found that the increase in the molecular weight of PEG from 400 to 20,000 Da has significant impacts on the number of pores, as well as the porosity of the resultant membrane, which led to enhanced hydraulic permeability. However, hydrophilic PEG is highly soluble in water and, thus, very likely to leach out during the phase inversion process. Because of this reason, there is a safety concern regarding the possible leaching of PEG from the membrane into the blood stream during hemodialysis. Such an issue could cause complications in renal failure patients.

To avoid leaching, amphiphilic copolymers containing vinylpyrrolidone (VP) chains could potentially be used. Some of the examples of VP-derivative terpolymers are poly(methylmethacrylate-co-acrylicacid-co-vinylpyrrolidone) (PMMA-co-AA-co-VP) and poly(acrylonitrile-co-acrylicacid-co-vinylpyrrolidone) (PAN-co-AA-co-VP) [93]. The existence of acrylonitrile or methylmethacrylate chains in the structure could inhibit the leaching of the hydrophilic polymer during operation. In addition, the variations in their molecular structures (e.g., linear, comb-like, dumbbell-like and chain-sphere-like structure) could achieve better resistance against protein adsorption.

Song et al. [94] synthesized a comb-like amphiphilic block copolymer poly (vinyl pyrrolidone)-block-poly (acrylate)-graft-poly (methyl methacryate)-block-poly-(vinyl pyrrolidone) (PVP-b-P(AE-g-PMMA)-b-PVP) and used it to modify PES membranes via the blending method. The results revealed that the hydrophilicity of the PES membrane was enhanced upon PVP-b-P(AE-g-PMMA)-b-PVP incorporation. This, as a result, improved the membrane pure water flux and its hemocompatibility. The result of the pure water flux was increased remarkably from 17.60 mL/m^2^.h.mmHg in the pristine membrane to 134.50 mL/m^2^.h.mmHg upon modification. Furthermore, the modified membrane showed good flux stability even after being immersed in water for 30 days prior to its performance assessment.

Li et al. [95] modified a PES membrane with citric acid-grafted polyurethane (PU) and found that the modified membrane displayed lower bovine serum fibrinogen (BFG) and BSA adsorption and was able to suppress platelet adhesion. Owing to the binding effect of calcium ions in the blood, the modified membrane prolonged the APTT (55 s) and PT times (17 s) of the unmodified membrane (APTT: 30.2 s and PT: 15 s). Furthermore, the modified membranes demonstrated a good cytocompatibility with the increased amount of hepatocyte cells grown on the membranes. This strongly suggested the positive features of citric acid-grafted PU for the development of an improved hemodialysis membrane.

In another study, a heparin-like structured macromolecule (HLSS) (poly(St-co-AA)-b-poly(VP)-b-poly(St-co-AA)) was synthesized by RAFT polymerization using carboxyl-terminated trithiocarbonate followed by adding it into a PES dope solution to fabricate a flat sheet membrane [96]. The presence of anionic functional groups (–SO_3_H, –COOH and –OH) on the membrane surface can repel coagulation factors in the blood, prolonging the time of coagulation. Nevertheless, the incorporation of >7 wt% of HLSM made the blood incoagulable. Wang et al. [97] synthesized an HEP-like PES (HLPES) containing sodium carboxylic (–COONa) and sodium sulfonic (–SO_3_Na) groups. The HLPES was self-synthesized through a combination of polycondensation and post-carboxylation methods followed by blending it with PES at various ratios. The HLPES-modified membranes presented lower platelet and BSA adsorption, prolonged TT and APTT and suppressed leukocytes and complement system activation, which can be attributed to the presence of–COONa and –SO_3_Na groups in the heparin-like structure. Apart from that, Beek et al. [44] modified PES hollow fiber membranes by blending them with small amounts of a random copolymer consisting of N-vinylpyrrolidone and N-butylmethacrylate, focusing on the long-term stability of the membrane and its removal against protein-bound toxins, i.e., hippuric acid (179 Da) and indoxyl sulfate (213 Da). The results showed that the fabricated membranes could achieve high removal for indoxyl sulfate (90%). However, for the smaller size hippuric acid, a lower removal rate (30%) was reported.

#### 4.2.2. Inorganic Materials

In recent years, the incorporation of inorganic nanoparticles to form nanocomposite membranes is receiving attention from researchers for various applications [98]. The presence of evenly dispersed and distributed inorganic nanoparticles in the polymer matrix has proven to enhance the performance of conventionally used polymeric membranes [99]. However, hemodialysis experiences a very precautionary transition from the current commercial membranes to the utilization of nanoparticle-modified membranes. To the best of our knowledge, there is no dialysis membrane in the current market that is modified by inorganic nanomaterials.

Very limited studies related to nanomaterial-modified hemodialysis membranes could be found in the literature. To date, only several types of nanomaterials, such as carbon-based nanomaterials, metal oxides and zeolites, were tested at the laboratory level to modify hemodialysis membranes [100,101]. Typically, nanomaterials possess several unique properties, including a large surface area, special functional groups and superior hydrophilicity. Some nanomaterials such as silver nanoparticles and iron oxide nanoparticles can develop a strong antimicrobial property that is important in improving membrane biocompatibility [102,103]. In this section, focus is placed on the roles of two types of nanomaterials, i.e., carbon-based nanomaterials and metal oxides, on the characteristics of hemodialysis membranes.

Irfan et al. [104] made the attempt to fabricate PES/multi-walled carbon nanotube (MWCNT) flat sheet membranes via the phase inversion process. The resultant PES/MWCNT membranes were found to be more hydrophilic, recording an enhanced pure water flux of 72.20 L/m^2^h (measured at 1 bar) compared to 7.14 L/m^2^h shown by the pristine PES membrane. In terms of uremic toxin clearance, the modified membrane achieved 56.30% urea clearance, 55.08% creatinine clearance and 27.90% lysozyme clearance. Using heparin-mimicking polymer brush-grafted CNTs as an inorganic additive, Nie et al. [105] developed new type of PES nanocomposite membrane. The membrane exhibited enhanced albumin separation (96.8% BSA rejection) with excellent blood and cell compatibility, and no obvious activation of the coagulation cascade occurred on the membrane.

**Figure 6:**
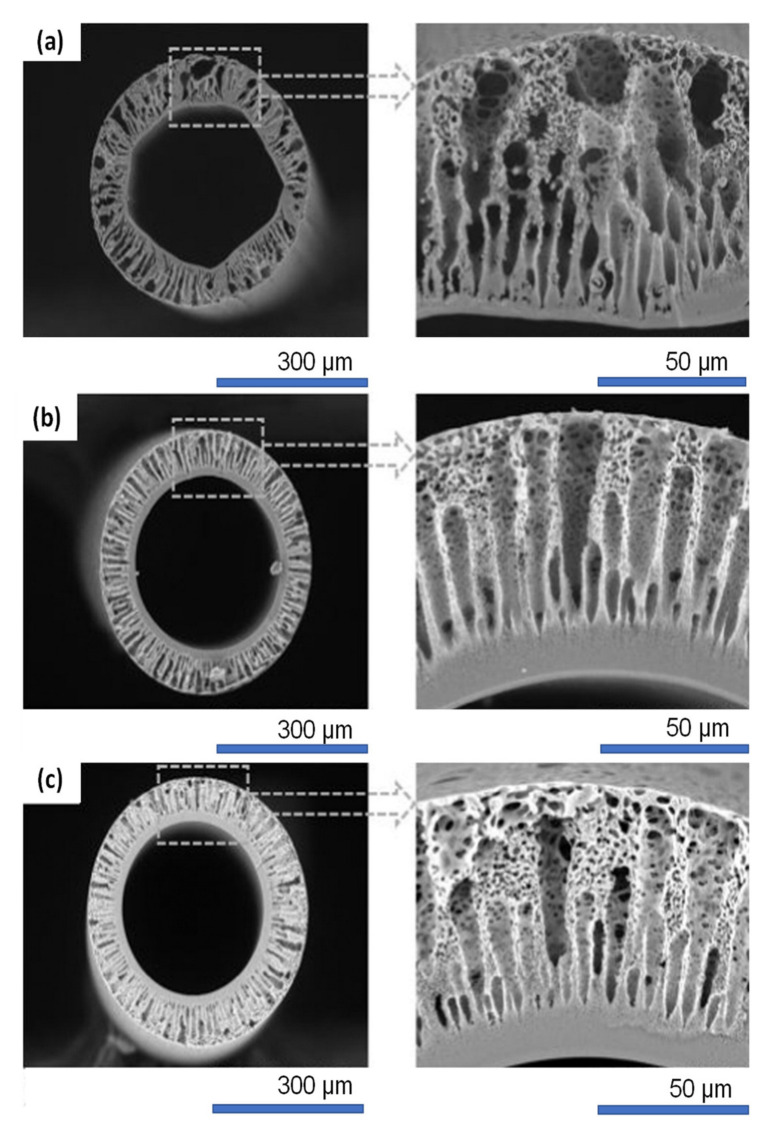
The microscopic images of (**a**) pristine PES, (**b**) PES/O-MWCNTs and (**c**) PES/PCA-g-MWCNT membranes [31].

The versatility of CNTs in improving hemodialysis membranes was further studied by Abidin et al. [31]. A high-performance PES/MWCNT membrane was synthesized by growing hyperbranched poly(citric acid) on the surface of MWCNTs followed by embedding it into the PES membrane matrix. In Figure 6, the finger-like structure of the PES membrane extends from the inner layer upon the addition of MWCNTs due to an accelerated phase inversion process. The skin layer at the innermost region of the modified membranes was found to be thicker as a result of the increase in the de-mixing rate of the solvent and the non-solvent during the phase inversion process. The PES/PCA-g-MWCNT membrane showed an outstanding separation performance (pure water flux of 95.36 L/m^2^h and BSA rejection of 95.2%) coupled with a good antifouling characteristic, achieving a flux recovery rate of 81%. As a comparison, the control membrane only showed 77.56 L/m^2^h pure water flux, 41.3% BSA rejection and 50% flux recovery rate [31].

Zare-Zardini et al. [106] evaluated the hazardous effects of arginine-treated MWCNTs (MWCNT-Arg) and silver nanoparticles on blood cells during hemodialysis. Silver nanoparticles are well-known antimicrobial agents, while the frictionless channel of MWCNTs with precise pore entries can facilitate the transport of water molecules. An in vivo study revealed that both MWCNT-Arg and silver nanoparticles tended to decrease the number of red blood cells and hematocrit and have a direct influence on white blood cell drop. The hemolytic rate of the membrane modified by silver nanoparticles (3.04%), however, was lower than that of the membrane modified by MWCNT-Arg (3.28%), revealing that silver nanoparticles have a lower toxicity compared to MWCNT-Arg.

Modi et al. [107] introduced 2D graphene oxide (GO) nanosheets into PES hollow fiber membranes and obtained better membrane properties in terms of hydrophilicity, hemocompatibility, cell attachment and separation performance. This is due to the remarkable interfacial compatibility between GO and PES. The GO-modified membrane also showed improved bioactivity to support the monolayer attachment, growth and proliferation of human kidney cells. The developed membrane showed excellent clearances against urea (85%), creatinine (70%) and phosphorus (67%). The removal of those uremic toxins was reported to be 1.6–3.3 times higher than that of a commercial PSf dialyzer (Hemoflow, Fresenius).

Xia et al. [108] modified a PES membrane by immobilizing it with antifouling silver-nanogels (Ag-nanogels). The results revealed that the pure water flux of the pristine membrane was significantly improved from only 13.5 to >400 mL/m^2^·h·mmHg upon the immobilization of Ag-nanogels due to the improved hydrophilicity of the membranes. With respect to separation, the modified membrane also showed higher BSA rejection (90%) than that of the pristine membrane (74%). From the platelet adhesion results, it was concluded that the presence of Ag-nanogels in the membrane was positive in reducing platelet adhesion. Furthermore, the PES/Ag-nanogel-modified membrane was able to prolong the APTT and PT times of the pristine membrane.

Among the metal oxide nanoparticles, iron oxide (Fe_3_O_4_) nanoparticles have attracted great interest due to their hydrophilicity, biocompatibility, chemical stability and nontoxicity as a contrast agent for in vitro diagnostics and biocatalysis. A study by Said et al. [109] revealed that Fe_3_O_4_ has the potential to improve PSf membranes for an enhanced separation of middle-molecular uremic toxins. However, the safety concern regarding the possible toxicity of Fe_3_O_4_ toward humans might restrict the applications of these modified membranes for hemodialysis treatment.

**Figure 7:**
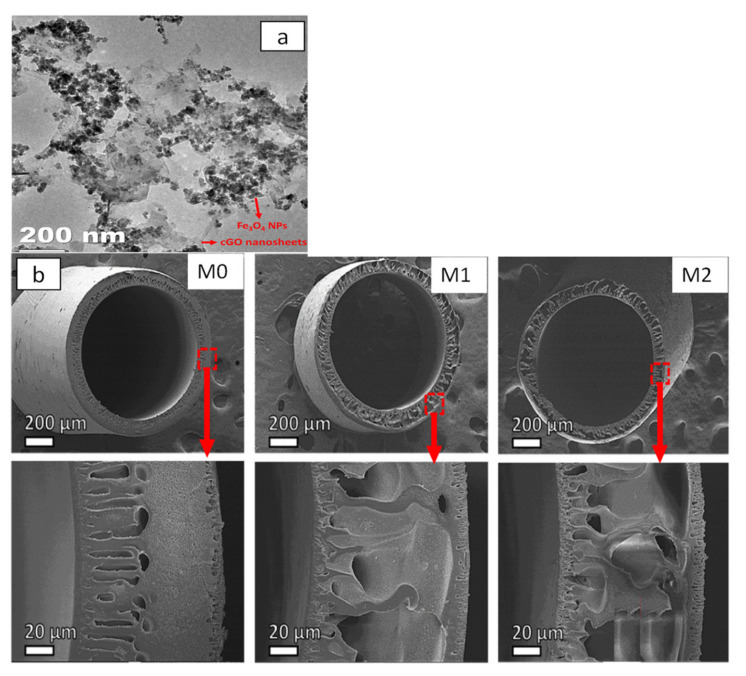
(**a**) TEM image of synthesized Fe_3_O_4_/cGO and (**b**) SEM images of hollow fiber membranes modified by different Fe_3_O_4_/cGO loadings (note: M0—pristine membrane, M1—0.05% Fe_3_O_4_/cGO and M2—0.10% Fe_3_O_4_/cGO) [110].

Modi and Bellare [110] incorporated iron oxide-decorated carboxylated GO (Fe_3_O_4_/cGO) into PES membranes for protein separation. The surface morphology of Fe_3_O_4_/cGO was studied using TEM, and the results (Figure 7a) showed that the Fe_3_O_4_ nanoparticles were uniformly dispersed over cGO nanosheets. Figure 7b shows that all the membranes exhibited asymmetric structures, which comprise a finger-like structure in the inner and outermost layers as well as sponge-like macropores in the middle part. Upon incorporation of 0.1 wt% Fe_3_O_4_/cGO, the resultant membrane demonstrated the highest pure water flux (110 L/m^2^·h at 0.5 bar) and was able to effectively reject different solutes, achieving 92.9, 94.5, 99.5, 100 and 100% rejection against lysozyme, trypsin, human serum albumin, human-γ-globulin and human fibrinogen, respectively. Th results also showed that the performance of the PES/ Fe_3_O_4_/cGO membrane was stably maintained over a 5 h testing period.

Over the years, we have seen many relevant studies carried out in order to improve membrane performance using different kinds of polymers, additives and fabrication approaches. Table 6 highlights the key findings of these studies.

### 4.3. Dialyzer Design

#### 4.3.1. Dialyzer Housing and Design

The housing material is an important aspect of a dialyzer. A good housing should be small in size to minimize transport and storage costs and be able to achieve optimal performance under various treatment conditions. In addition, a dialyzer housing should have a good transparency, good mechanical stability and be able to withstand different sterilization conditions. Moreover, it is critical to ensure that the materials used for making the housing do not release any substances into the dialysate and blood. Currently, dialyzer housing materials are manufactured mainly from polycarbonate (PC) or polypropylene (PP) by injection molding [41].

Polycarbonate is considered the golden standard due to its excellent mechanical stability and unique material characteristic. However, based on the current findings, PC housing could cause an elution of bisphenol A (BPA), leaching it into blood during dialysis [16]. The presence of BPA in the body might increase the risks associated with cancer and other congenital disorders. Therefore, eliminating this polymer from the housing material is extremely important. Currently, some dialyzers such as the FX-class dialyzer (Fresenius, Germany) are made of PP housing because PP can be disposed in an environmentally friendly manner [26].

The dialyzer basically consists of three main plastic parts, namely the body housing (for fiber bundles) and two endcaps [113]. Endcaps are used to ensure an equal flow distribution of blood and dialysate during dialysis. The body design of a dialyzer is of major importance to maximize solute transfer and the dialyzer mass transfer area coefficient [14,114]. The density of the fibers inside the housing could affect the resistance of the dialysate flow and further its flow within the fibers [41]. It has been reported that an increase in the fiber packing density from 45% (standard) to 60% will increase the ratio between the bundle volume and the internal volume of the housing [26]. Thus, the priming volume of the dialysate will reduce, causing an increase in the average velocity of the dialysate in the housing [114]. As a result, an improved effect on counter current flow conditions and an enhanced mass transfer coefficient could be attained. In the FX-class dialyzer, a different approach based on special undulation coupled with micro-crimp fiber geometry was used to increase the fiber packing density from 45 to 60% [26]. Such an approach is effective to prevent single fibers from coming close to one another.

In addition, the dimension and the number of fibers are other factors that can influence the hydraulic resistance of the dialyzer. For the high convection rates, an equilibrium filtration pressure may occur along the length of the dialyzer due to increasing viscosity of plasma blood. Thus, the pressure inside the fiber lumen is higher compared to that of the dialysate side of the dialyzer [27]. It must also be pointed out that by reducing the wall thickness of the fibers packed within the dialyzer from 40 to 35 μm, one study revealed that the modified dialyzer could achieve improved small-molecular clearance without any loss of mechanical stability [115]. Another study also showed that by reducing the internal fiber diameter by 7.5% (from 200 to 185 μm), it could increase the pressure drop by approximately 25%, leading to higher internal filtration and an improved middle-molecular clearance [26].

#### 4.3.2. Arterial Port

**Figure 8:**
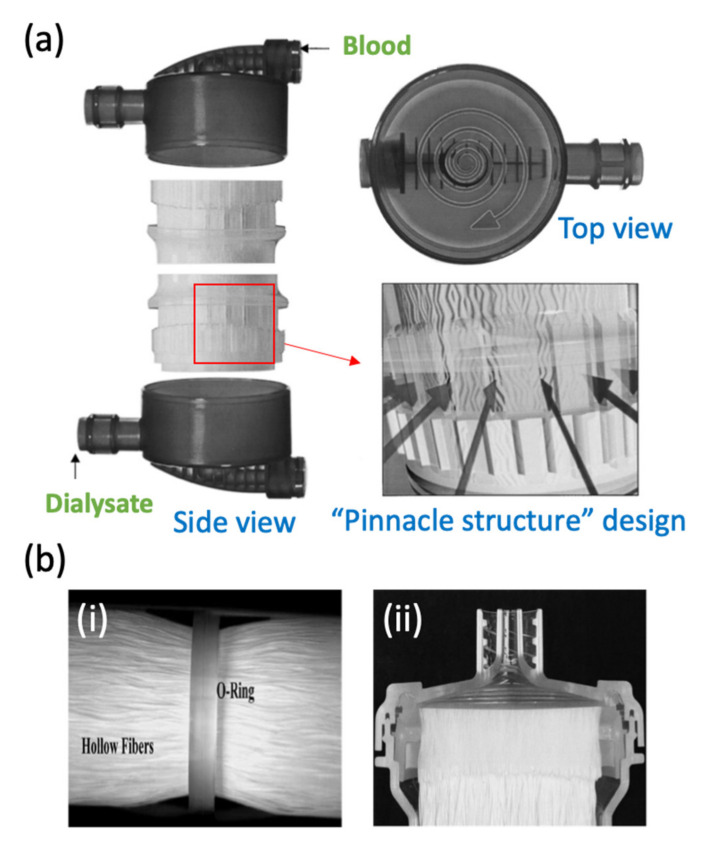
(**a**) Location of blood port and dialysate port of a dialyzer (FX-class hemodialyzer, Fresenius) with “pinnacle structure” design [26]. (**b**) (**i**) The location of O-ring in the middle part of dialyzer and (**ii**) cross-section of 2.2-m^2^ dialyzer (Fresenius Polysulfone^®^) with housing made of PP [113].

Ideally, the arterial port (also known as the blood port) must have minimal flow stagnation to guarantee a homogeneous flow distribution for all the fibers. For this purpose, various types of flow distributors (e.g., conical and spiral distributors) have been proposed with the aim of reducing the space between the cap and the potting [25,113]. The blood ports are designed in order to distribute the blood in a spiral configuration in the potted fiber region compartment as shown in Figure 8a [26]. Together with a specific ring seal, the design could reduce the dead spaces and further decrease the blood trauma that normally occurs when blood enters the dialyzer [116]. It must be noted that an optimized blood port together with a well-designed spatial distribution of the fibers (in the potting area) could lead to a homogeneous distribution of the dialyzer. The lateral blood inlet port design can offer a homogeneous blood flow in the dialyzer header, preventing stagnation zones in the dialyzer [26,114].

#### 4.3.3. Dialysate Port

The dialysate compartment and port are designed to achieve uniform flow distribution within the dialyzer. Previous studies demonstrated that when the dialysate flow rate of standard dialyzers was set beyond 600 mL/min, the clearance of dialyzers against small-molecule toxins was negatively affected [26,30]. This problem can be explained based on the channeling phenomenon that develops a preferential flow in the dialysate (in the region external to the bundle), causing consequent stagnation in the internal region of the dialyzer. To address this issue, an overflow ring (O-ring) could be introduced in the dialysate compartment as shown in Figure 8b [113]. The presence of a pinnacle structure on the dialysate ports enables the homogeneous distribution of flow.

A study conducted by Ronco et al. [26] showed that the pinnacle design was able to improve the dialysate distribution. In this work, dye solution was injected in the dialysate compartment and analyzed by computerized helical scanning, aiming to monitor the flow distribution within the compartment. The authors reported that no major discrepancies in flow velocity were detected from the periphery to the central region of the bundle. This is mainly attributed to the specific micro-undulation of the hollow fiber membranes that prevented dead zones and channeling phenomena.

For the conventional dialyzer, dialysate typically enters directly through the dialysate port into the actual fiber compartment. This, however, could lead to an inappropriate dialysate distribution, causing poor coverage or large parts of the fiber bundles. Thus, the pinnacle structure of the housing could act as a dialysate distributor and keep the flow rate of the dialysate entering from all sides into the fiber bundles consistently.

#### 4.3.4. Potting Material

To separate the dialysate from the blood compartment, proper potting must be performed on the hollow fiber membranes. Both ends of the fibers are required to be glued permanently to the housing using safe potting material, i.e., polyurethane (PU). Compared to other potting materials, such as silicone elastomers and epoxide resins, PU is proven to be safe by not releasing toxicological products during hemodialysis [117].

**Figure 9:**
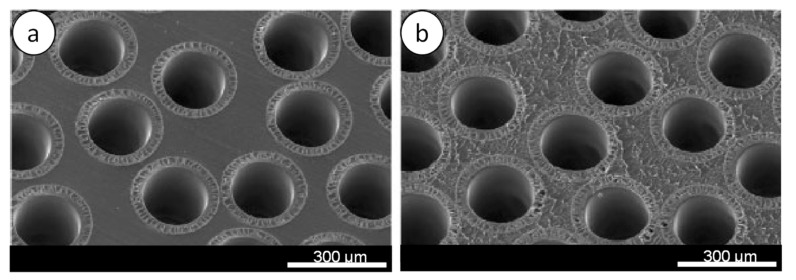
SEM images of the cutting surfaces of two dialyzers, (**a**) smooth blood contacting surface and (**b**) rough blood contacting surface [118].

Typically, several thousands of hollow fibers are arranged and packed as a bundle in a dialyzer. After the fiber bundle is inserted into the plastic cylinder about one foot long, the fibers are sealed using PU to encapsulate the fibers. The bundle is then cut with a specialized blade at a defined temperature after the PU is cured. Despite the biocompatible property of PU, there is a potential risk for it to cause coagulation at the blood contacting surface consisting of open fiber ends. Thus, the cutting surface of the dialyzer must be very smooth and free from defects. Figure 9a shows examples of an acceptable cutting surface of two dialyzers, whereas Figure 9b shows a cutting surface that is too rough [115]. Rough surfaces can be caused by an improper cutting technique, and, if this happens, the dialyzer should be discarded.

Currently, centrifuge systems are widely applied to perform potting, and the plotting conditions must be carefully adjusted according to the hollow fiber dimension, fiber porosity and stability. The ratio of two components to form PU, i.e., polyol and isocyanate, must be optimized to achieve the desired hardness for the potting and to meet the requirements of the sterilization process. Currently, a knife-based system is mainly applied for the cutting procedure for a smooth cutting of the dialyzer surface [117].

#### 4.3.5. Sterilization Process

Sterilization is important in the production line of dialyzers to destruct all forms of pathogens to ensure safe use. In the past, dialyzers were assembled and washed with alcohol before clinical use. A gas plasma sterilization system using hydrogen peroxide (H_2_O_2_) was practically used in dialyzer production but stopped in the late 1980s due to adverse effects on patients [119]. Currently, there are many sterilization techniques available (see Table 1), including the traditional methods of autoclaving, gamma irradiation and ethylene oxide (EO). Low-temperature gas plasma and vapor phase sterilant are the newly introduced sterilization processes.

Prior to the selection of a sterilization method, the compatibility of the membrane with the sterilization conditions and the chemicals used must be known first. PSf-based dialyzers, for instance, are dialyzers that can withstand various sterilization conditions owing to its high thermal resistance (150–170 °C), good pH stability, resistance to oxidative medium (5–7% hypochlorite and 3–5% H_2_O_2_) and high mechanical stability against torsion and fracture.

Elrlenkötter et al. [120] conducted an in vitro assessment of the hemocompatibility pattern of five different dialyzers (Toraysulfone, Toray; PEPA, Nikkiso; Helixone^®^, Fresenius; Cuprophan, Gambro and Hemophan, Gambro) at two different modes of sterilization, i.e., steam sterilized and gamma-ray sterilized. A total hemocompatibility score (THS) was then calculated by measuring five different hemocompatibility parameters, i.e., plasmatic immune systems, coagulation activation, cell activation, platelet count and platelet factor. The researchers found that the THS of the steam-sterilized PSF membrane (Helixone^®^, Fresenius) was 19.6, i.e., much lower compared to that of the gamma-sterilized PSf membrane from Toraysulfone, which is 32. The lower the THS the better the hemocompatibility degree and vice versa [121]. It must be noted that the factors that affect the hemocompatibility pattern of a dialyzer, such as potting material, sterilization technique and membrane geometry, could also influence the hemocompatibility profiles of the dialyzer [120].

**Figure 10:**
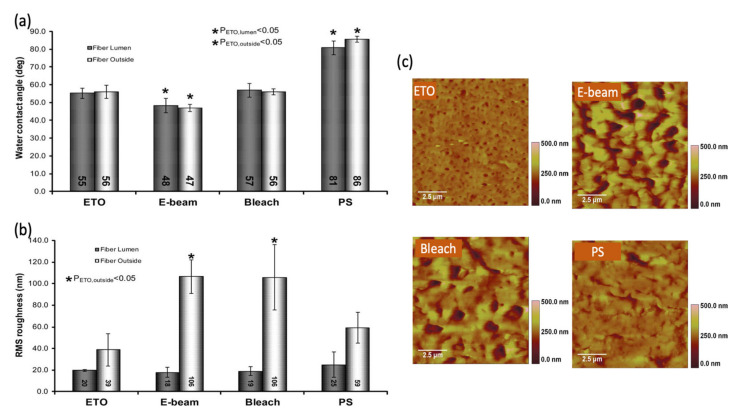
(**a**,**b**) Water contact angle and roughness of the inner (lumen) and outer surface of PSf membranes sterilized by different methods and (**c**) AFM images of outer surface of different fibers [119].

Madsen et al. [119] observed significant changes in the physiochemical properties (hydrophilicity and morphology) of a commercial PSf dialyzer (Optiflux, Fresenius) after subjecting it to different methods of sterilization, i.e., standard ethylene oxide (ETO), electron beam (EB) and sodium hypochlorite (bleach) sterilization. In Figure 10a, the PSf membranes with EB sterilization exhibit the greatest hydrophilicity (i.e., lowest water contact angle) compared to the ETO-sterilized and bleach-treated fibers. This indicated that the post-treatment selected for the dialysis membranes could have different outcomes and should be carefully evaluated. In certain cases, it altered membrane properties, such as permeability and pore size [122,123]. With respect to surface roughness (Figure 10b), no significant difference was found on the inner surface (lumen side) of different membranes. However, both bleach-treated and E-beam-sterilized fibers exhibited a significantly higher roughness value on their outer surfaces compared to the ETO-sterilized and PS fibers. The AFM images shown in Figure 10c clearly indicate that the morphology of the PS fiber was altered after undergoing bleach and E-beam sterilization. This was likely due to polymer restructuring associated with sterilization techniques. Such a morphological change could alter solute transfer and weaken the mechanical property of the fiber [124,125].

Togo et al. [126] compared the effects of gamma-ray sterilization and autoclave techniques on the biocompatibility of two commercial PSf dialyzers, namely APS-11SA (Asahi Kasei medical Co.) and RENAK (Kawasumi). The results showed that no platelet adhesion was observed on the APS-11SA membranes, while for the RENAK membranes, only a little platelet adhesion was observed. However, the APS-11SA dialyzer showed excellent blood compatibility compared to the RENAK dialyzer. This is because its PVP (added into the membrane matrix) was insolubilized by cross-linking using gamma rays [127]. Since the RENAK dialyzer was sterilized with an autoclave, an excess amount of PVP could be eluded during the rinsing procedure.

Yamashita et al. [30] compared the impacts of different sterilization methods (i.e., autoclave sterilization (AC), gamma-ray sterilization and AC+gamma-ray sterilization and AC+AC) on the performance of super high-flux PSf dialyzers (Kawasumi, Japan) with respect to solute transport (e.g., creatinine (113 Da), vitamin B_12_ (1355 Da) and sieving coefficient for albumin (66.7 kDa)). The results showed that without any sterilization, the dialyzer showed the lowest clearances against creatinine and vitamin B_12_. However, there was no significant change in solute transport for the dialyzers treated with different sterilization methods. Comparing the sieving coefficient of albumin, the highest value was found in the dialyzer with AC, followed by AC+AC, AC+gamma-ray, gamma-ray sterilization and no sterilization. It was quite clear that the sterilization process could enlarge the membrane pores, which increase the solute transport rate. Table 7 further compares the conditions of different sterilization techniques employed for dialyzers.

### 4.4. In Vitro Performance

An accurate solute transport prediction of a dialyzer is essential when prescribing a dialyzer to patients. The transport characteristics of dialyzers are often first examined in vitro, and such data are usually provided by dialyzer manufacturers. The clearance data provided by the manufacturers are obtained from in vitro experiments using water, but the value is always higher than the blood clearance obtained in vivo, as water contains no foulants. The typical criteria used to evaluate dialyzer transport include the *K_UF_* and the clearances of small- and middle-molecule toxins. The *K_UF_* is important when using dialysis machines that do not provide an automatic volumetric control, and it partly determines the amount of back filtration across the dialysis membrane [66].

The performances of selected commercial PSf dialyzers (with the same effective surface area of 1.8 m^2^) are highlighted in Table 6. Clearance may be considered the most important characteristic of a dialyzer because it is a critical factor in determining the dialysis prescription [12,131]. In view of this, urea clearance is the most common marker to define the quality of a dialyzer. The clearance of creatinine and phosphate can also be used as indicators to evaluate the performance of a dialyzer in removing small-molecular toxins.

It can be observed in Table 6 that the removal of small-molecular toxins is not significantly different between REXEED, Revaclear-400, FX-80 and Toraysulfone dialyzers. The diffusion of small-molecular toxins in the microporous membrane is determined based on the pore size and its distribution in the membrane skin layer. For this diffusive clearance, increasing both the dialysate and flows can increase the concentration gradient clearance while maximizing contact between the dialyzer and blood surface.

Larger size uremic solutes are better separated by convective clearance in comparison to diffusion using the same dialyzer. In order to increase convective clearance, a balance must be achieved between a larger internal diameter of the fiber (to allow greater convective movement while maintaining the portion of blood plasma to be filtered) and a smaller fiber diameter (to generate higher hydrostatic pressure needed to drive convection). Vitamin B_12_ (molecular weight 1355 Da) has a lower clearance and helps in defining the permeability of the dialyzer for larger (middle) molecules. Recently, β_2_-M clearance has also been used as a method to assess membrane characteristics, particularly the flux of the membrane [39,64].

The *K_o_A* is typically used to determine the permeability of the mass transfer between the blood and the dialysate pathway of the dialyzer [132]. From the table, Revaclear achieves the highest *K_o_A* value followed by REXEED, FX80 and Toraysulfone. It must be noted that the different conditions employed in manufacturing the dialyzer could lead to variation in the *K_o_A* value.

The *K_UF_* value as shown in the table represents the quantity of the fluid flow across the dialysis membrane, which is related to the transmembrane pressure. Among the four high-flux dialyzers highlighted, REXEED shows the highest *K_UF_* value followed by FX-80. A high-flux dialyzer is typically defined based on a β_2_-M sieving coefficient (i.e., >0.6) and can potentially separate solutes between 10 and 50 kDa. Ideally, large molecules are easily removed by convection at a high *K_UF_* value. However, such an approach is not practical, as kidney disease patients cannot tolerate too high a K*_UF_* value.

The ability of a dialyzer to sieve or reject solutes during dialysis has been known as a marker for the clinician to determine the suitable dialyzer for patient [116]. The sieving coefficient is between 0.9 and 1.0 for inulin (5.2 kDa), 0.9–1.0 for β_2_-M (11.8 kDa), 0.1–0.4 for myoglobin (16 kDa) and <0.001 for albumin (66.5 kDa) for the selected commercial PSf dialyzers shown in Table 8. Generally, there is no significant difference between these commercial dialyzers. The high sieving coefficient for β_2_-M and myoglobin is a good indicator to determine the high removal of middle-molecular toxins during hemodialysis [20,109].

### 4.5. Flow Simulation

The flow distribution in blood and dialysate compartments has a large impact on the mass transfer efficiency of the membrane. Any mismatch caused by non-uniform flow in either the blood or dialysate compartment could lead to a lesser uremic solute removal from the blood [133]. Filtration experiments can be performed to obtain the optimal flow for the dialyzer, but such an approach is time consuming. In view of this, computer simulation offers a solution to predict the optimal operating conditions of dialyzers.

**Figure 11:**
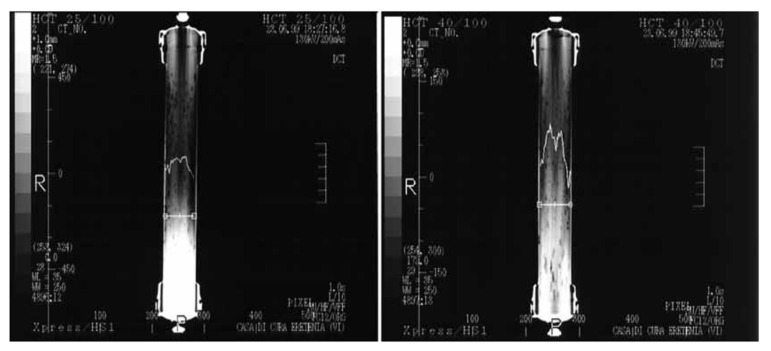
Selected images captured for the conditions of 25% Hct (**left**) and 40% Hct (**right**) in the blood compartment [113].

Ronco et al. [25] carried out a study to investigate blood and dialysate flow distributions (in vitro) in dialyzers using the computerized helical scanning technique. Blood flow distribution was investigated by injecting dye into the blood compartment using human blood with 25 and 40% hematocrit (Hct). Sequential images were then captured from the helical scanner. The average and regional blood flow velocities together with wall shear rates were then determined using the reconstructed imaging sequence. The densitometric profile describing the blood flow distribution is presented in Figure 11. As shown, the parabolic bimodal shape is more significant in the images captured at 40% Hct, indicating a further alteration of the flow distribution. In short, blood was found to be distributed non-proportionally in the dialyzer and is strongly affected by the Hct level. From the reconstructed imaging sequence, this allows the calculation of the single-fiber blood flow and the single-fiber wall shear rate at different regions of the dialyzer. This phenomenon is due to the remarkable reduction in plasma water flow across the dialyzer in the presence of a progressive Hct rise. Another possible explanation for this phenomenon is when the Hct level is high, the single-fiber blood flow velocity and the single-fiber wall shear rate are obviously lower compared to the fiber found at the periphery of the bundle. It must also be noted that the single-fiber wall shear rate was likely to decrease in peripheral fibers, presenting a value near half of that observed in the central fiber of the bundle.

**Figure 12:**
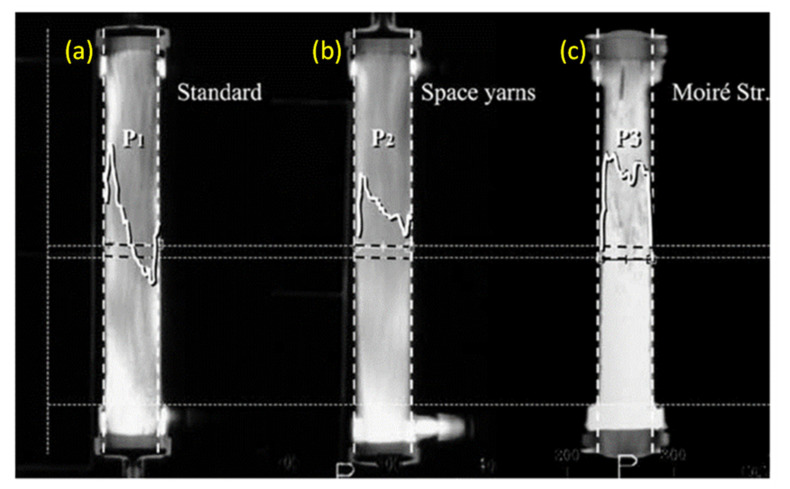
Contrastographic images of three different hemodialyzers, (**a**) P1—standard dialyzer, (**b**) P2—spacer yarn design dialyzer and (**c**) P3—Moirè structure [113].

A similar technique was also employed to access the flow distribution in the dialysate compartment in three different designs of dialyzers, i.e., a standard configuration, the Moirè structure (wave-shaped fiber) and spacer yarns (spacing filaments preventing contact between fibers) [25]. Figure 12 shows the contrastographic images using the helical scanner and dye injection. As shown, the most homogeneous distribution is observed in the case of the Moirè structure. Another study reported that hollow fiber dialyzers containing spacer yarns have better dialysate flow distributions compared to the dialyzers without spacer yarns and could achieve excellent clearance characteristics [134]. Changing the hollow fiber shape into wavy or undulating forms has shown improved flow distribution in in vitro experiments. It has been postulated that the presence of more wave-shaped hollow fibers tended to result in secondly or traverse flow across the individual fibers, enhancing mass transfer [134,135].

Eloot et al. [136] employed CFD to analyze flow patterns in a dialyzer and successfully developed a 3D microscopic model to simulate the blood and dialysate flow pattern of the low-flux PSf F6HPS dialyzer (Fresenius, Germany). The simulation results revealed the existence of homogeneous blood velocity over the complete radial section. Due to the boundary layer separation at the point of channel divergence and the impact of the inflowing blood on the inlet with a velocity in the range of 300–400 mm/s, vortices developed in the inlet manifold, which created a stagnant fluid layer. The simulation also indicated that deviation from a linear pressure drop–flow relationship is negligible for flow in dialyzers with a limited active length.

Nakasima et al. [137] investigated dialysate flow patterns using a longitudinal computed tomography (CT) scanning of two PSf dialyzers, i.e., APS-S (Asahi Medical) and TS-UL (Toray Medical). For a clinical comparison, an in vitro experiment was conducted after confirming the steady-state flow of fresh dialysate (500 mL/min) containing 5% BaSO_4_ and mock blood (xanthan gum solution; 200 mL/min). From the CT images, the dialysate distribution of the TS-UL dialyzer was observed to be homogenous, whereas it was not homogenous for the APS-S dialyzer. The albumin loss and the clearance of urea nitrogen of TS-UL were reported to be significantly higher compared to the APS-S dialyzer, and this could be due to the design differences in the dialysate compartment.

## 5. Technical Challenges of Dialyzer Development

Despite the significant progress in the technology and R&D activities, there are still many technical challenges associated with the advanced dialyzer development. One technical challenge is how to improve the performance of the dialysis membrane so that it can reduce the hemodialysis treatment time and its treatment frequency. Currently, a single session of conventional hemodialysis treatment requires 3 to 5 h to complete, and ESKD patients need a minimum of three treatment sessions per week according to the guidelines and recommendations of the National Kidney Foundation [138]. Shortening the treatment time with the use of the same type of dialysis membrane could lead to poor treatment outcomes for patients in the long term, including protein–energy malnutrition, amyloidosis and cardiovascular diseases [11]. In view of this, one strategy to address this issue is to optimize dialyzer efficiency by increasing its flux (>40 mL/h/mmHg) without compromising its urea clearance (maintained at least 60%). However, Himmelfarb et al. [8] reported that all-cause and cardiovascular mortality for patients on dialysis are not clearly reduced despite increasing the dialyzer flux. Thus, focus should be placed on improving dialysis efficiency and patients’ outcomes instead of reducing treatment duration.

In terms of material development, a significant amount of research articles have shown the potential of using inorganic nanomaterials in improving the performance of membranes (particularly MWCNT and GO) at the laboratory level [107,139,140], but none of the studies have demonstrated the long-term stability of nanomaterials in the membrane matrix or included the results of clinical tests for hemodialysis treatment. Currently, there is also no dialysis membrane on the market that is modified by inorganic nanomaterials. Researchers believe that the advances in nanotechnology may pave the way to the mass manufacture of more selective membranes for hemodialysis [141] but bear in mind that the cost and cost effectiveness of the dialysis membrane and the dialyzer are important considerations for patients. Worldwide, a substantial number of people in many low- and middle-income countries are still not able to afford hemodialysis treatment and depend heavily on the subsidies provided by the governments.

The usage of harmful organic solvents during membrane fabrication has also prompted a move away from harmful solvents to green alternatives to achieve sustainability. However, an ongoing challenge appears while replacing traditional solvents with green solvents. This is mainly due to the higher cost of green solvents and the limited choices available on the market for membrane fabrication. Furthermore, the performance of the resultant membranes (UF type) during the hemodialysis process also remains largely unclear, as almost of all of the membranes synthesized in the laboratory setting using green solvents are demonstrated for water applications.

Compared to the conventional dialysis membranes, double-layer mixed matrix membranes, which combine adsorption and dialysis, have demonstrated better results in terms of protein-bound uremic toxin removal [142]. However, these mixed matrix hollow fiber membranes were obviously large in diameter (500–700 μm) compared to the currently used membranes in clinical practice (200 μm) [20]. This, as a result, reduces the membrane packing density (m^2^/m^3^) in the dialyzer housing and affects the performance of the dialyzer.

**Figure 13:**
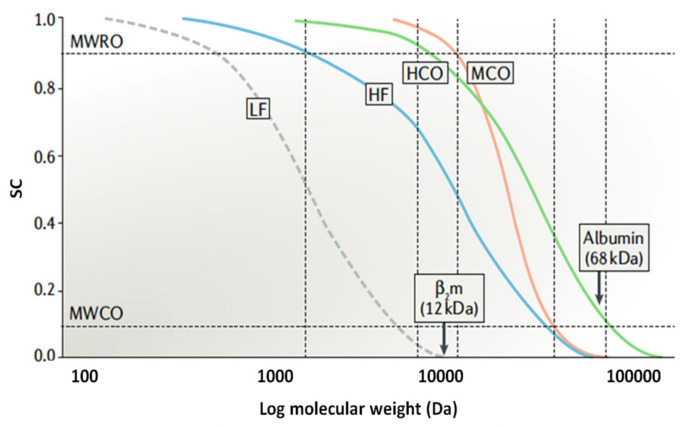
Theoretical sieving curves for 4 different classes of membranes: (1) low flux (LF), (2) high flux (HF), (3) high cut-off (HCO) and (4) medium cut-off (MCO). The point in the curve where the sieving coefficient (SC) is 0.9 determines the molecular weight retention onset (MWRO) value, while the point where SC is 0.1 determines the molecular weight cut-off (MWCO) value. As the interval between MWCO and MWRO decreases, the profile of the curve becomes steeper, which leads to increased removal of large uremic toxins (e.g., β_2_-m) but decreased albumin loss [24].

The new generation of dialysis membranes known as MCO membranes (also known as high-retention-onset (HRO) membranes) is reported to have an improved clearance of middle molecules [143]. Examples of MCO membranes are Theranova 400 and 500 from Baxter. These kind of membranes can effectively remove large middle molecules (up to 45 kDa by diffusion) by providing expanded hemodialysis (HDx). The concept of HRO is based on the steep curve between the molecular weight retention onset (MWRO) and the molecular weight cut-off (MWCO) achieved by a narrow distribution of membrane pore size. The additional MWRO parameter can bring out a second dimension of the membrane structural properties so that both the pore size and pore size distribution are fully considered in order to avoid unwanted misperceptions. Compared to the HCO membrane (see Figure 13), the MCO membrane is more capable of preserving the β_2_m sieving characteristics and able to improve the clearance of other large solutes (e.g., free light chains) while demonstrating a marked reduction in the permeability of albumin. What seems to be lacking at present is whether this expanded clearance of middle molecules can translate into reduced dialysis complications and better long-term patient survival.

In terms of dialyzer design, more R&D activities are still required to improve the distribution of the dialysate and blood flow in the dialyzer. Stagnation occurs in the flow channels of the blood and/or dialysis fluid as a result of concentration differences and could decrease the driving force of diffusion, negatively affecting dialysis efficiency. A study indicated that many factors could influence the flow in the dialyzer [14]. These include the hollow fiber shape, hollow fiber packing density, header shape and spacer yarns. Thus, a more sophisticated design can be introduced to optimize the performance of dialyzers. As an example, a unique circular wall header that fits into a circular groove in the hollow fiber bundle was previously designed by Fresenius for the blood port and has been incorporated into FX dialyzers to improve not only the blood flow rate but also the blood distribution in the lumen [134].

There is still room for improvement in relation to the biocompatibility of membranes. A biocompatible membrane could ensure the least amount of inflammatory response in patients during hemodialysis treatment. One practical approach includes treating the surface of dialyzers with a heparin and Hydrolink™ NV hydrophilic polymer [52]. Both materials are able to increase adsorbed water at the membrane–blood interface, improving antifouling and anti-thrombogenic effects [54]. Another material that is used to suppress biological responses is positively charged polyethyleneimine (PEI). This polymeric material is commercially used to neutralize the inner surface of a hollow fiber membrane (AN69-ST, Baxter) [144] to improve its biocompatibility. Kokubo et al. [145] elucidated that highly biocompatible dialysis membranes can only be developed when the overall correlations among biological reactions are thoroughly analyzed by integrating all data on biological responses elicited by mutual blood cell interactions and membrane–blood interactions. It is also important to note that both chemical and physical properties of the dialysis membranes are crucial and must be optimized during the surface modification process as a strategy to improve membrane biocompatibility. Variation in the membrane physical properties, such as roughness and softness, upon surface modification was found to alter the effect on leukocytes on platelets [145].

Lastly, we discuss the environmental impacts of using single-use dialyzers and dialysis membranes prepared from petroleum-based synthetic polymers (e.g., PSf and PES). Although biodegradable PLA seems to be a good candidate for dialysis membrane synthesis and has been researched over the years [80,81], its hydrophobic nature and low degree of biofouling resistance require it to be further modified through the incorporation of amphiphilic block polymers so that its hemocompatibility and antifouling performance can be enhanced for hemodialysis application. It is still unclear if the use of biodegradable PLA and the introduction of block polymers could lead to cost savings or at least be cost competitive compared to the typically used PSf and PES membranes. It might also be considered to offer dialyzer reuse as another opportunity to reduce solid waste disposal. It was reported that dialyzer reuse could eliminate the production of up to 46 million dialyzers in a single year, reducing the quantity of medical waste by more than 62 million pounds [146]. Nevertheless, Upadhyay et al. [147] reported that there was a decline in reusing dialyzers in the USA between 1997 and 2002, and the trend has further declined owing to the change in practice patterns with some dialysis providers favoring single use. Although cost saving is clearly driving the practice of dialyzer reuse, concerns associated with the increased health hazard from germicide exposure and disposal are raised [4]. Furthermore, it is possible for human errors to occur during dialyzer reprocessing, leading to infection transmission. This could increase the mortality risk with dialyzer reuse and potentially result in legal liability.

## 6. Conclusions

Dialyzers have become a core product for the treatment of patients with AKI and ESKD. The increased number of kidney disease patients of different stages worldwide and the elevated economical constraints in the healthcare environment have motivated membrane manufacturers to compete to produce advanced dialyzers at affordable prices. This article provides an up-to-date comprehensive review on the development of commercial and laboratory dialyzers for hemodialysis application and discusses the technical aspects of dialyzers, including dialysis membranes, dialyzer design, sterilization and flow simulation. Although dialyzers have been commercially used since the 1950s without significant technical drawbacks, the progress in improving their efficiency has never stopped over the past decades. In fact, we have seen membrane manufacturers launch a new generation of dialyzers that aim to improve hemodialysis treatment. These include a super high-flux dialyzer, an MCO dialyzer, a heparin-coated dialyzer and a vitamin E-coated dialyzer. These new generation dialyzers improve not only the quality of the filtered blood but also the patients’ quality of life and their survival rate. Nevertheless, the current state of technology still needs more R&D to achieve a greater dialyzer performance. Some issues that remain to be solved are the biocompatibility of dialyzers, the maldistribution of blood and dialysate flow, the high cost of new-generation dialyzers and the relatively wide membrane pore size distribution. We hope this review article can provide insights to researchers in developing/designing an ideal dialyzer that can bring the best hemodialysis treatment outcomes to kidney disease patients.

## Figures and Tables

**Table 1:** Commercial dialyzers in the current market.

Country	Dialyzer Series Name	Brand	^a^ Polymeric Material(s)	Sterilization
Germany	FX-class	Fresenius	PSf (Helixone)	Inline steam
	F-series		PSf	Inline steam
	Hemoflow™		PSf	Ethylene oxide, steam or electron beam
	Purema	Membrana	PES	Gamma ray
The United States of America	Polyflux L	Baxter	PAES, PVP and PA	Steam
	Theranova		PAES and PVP blend BPA-free	Steam
	Revaclear		PAES and PVP blend BPA-free	Steam
	Xevonta	B Braun	PSf	Gamma
	Diacap Pro		α PSf pro	Oxygen free gamma
Japan	ELISIO S	Nipro	PES (polynephron)	Gamma ray
	Sureflux		CTA	Gamma ray
	Solacea^TM^		CTA	Oxygen free gamma
	APS-U	Asahi	Asahi PSf	Gamma sterilized wet type
	ViE Series		Vitamin E-coated PSf	Gamma sterilized wet type
	Rexeed Series		PSf	Gamma ray and
	KF-201 Series		EVAL	Gamma ray
	Toraysulfone TS	Toray	PSf	Gamma ray
	Filtryzer		PMMA	Gamma ray
	Renak	Kawasumi	PSf	Gamma ray
China	F15	WEGO	PSf	Gamma ray
	HF15		PSf	Gamma ray

^a^ BPA (bisphenol A); CTA (cellulose triacetate); EVAL (ethylene vinyl alcohol copolymer); PA (polyamide); PAES (polyarylethersulfone); PES (polyethersulfone); PMMA (polymethylmethacrylate); PSf (polysulfone); PVP (polyvinylpyrrolidone).

**Table 2:** Classification of commercial dialyzers.

Class	^a^ Ultrafiltration Coefficient, K_UF_ (mL/h/mmHg)	β_2_-Microglobulin (β_2_-M)		Albumin		Ref
		^b^ Clearance (mL/min)	^c^ Sieving Coefficient	^d^ Loss into Dialysate (g)	^c^ Sieving Coefficient	
Low flux	<10	<10	-	0	0	[24]
High flux	20–40	20–80	<0.7–0.8	<0.5	<0.01	[24]
Medium cut-off	40–60	>80	0.99	2–4	<0.01	[28]
Protein leaking	>40	>80	0.9–1.0	2–6	0.01–0.03	[28]
Super high flux	40–60	-	1.0	9–23	<0.2	[29]

^a^ The coefficient is a specific property that characterizes a “clean” membrane, i.e., unfouled membrane. ^b^ For conventional hemodialysis with a blood flow rate of 200–400 mL/min. ^c^ In vitro for 1.5 m^2^ dialyzer. ^d^ For 4 h conventional hemodialysis.

**Table 3:** Types of uremic toxins in human blood.

Type of Uremic Toxin	Molecular Size	Descriptions	
Small water-soluble molecules 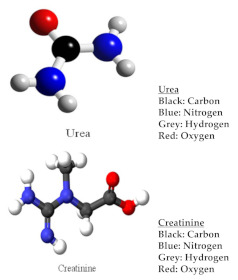	<500 Da	- Examples: urea (60 Da) and creatinine (113 Da). These two molecules are usually used as an indicator for the kidneys’ conditions [51]. - The most common uremic toxins in blood. - Can be removed easily via diffusion [52]. - There is a total of 68 small water-soluble uremic toxins out of 90 [51]. - The clearance is measured to estimate the performance and efficiency of treatment [53].	
Middle molecules 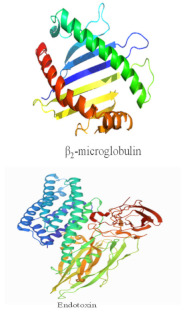	500–15,000 Da	- Examples: vitamin B_12_ (1355 Da), endotoxin fragment (1000–15,000 Da) and β_2_-M (11,818 Da) [18]. - This class of toxins is essentially peptides, which covers 24.4% of total uremic toxins [54]. - A total of 54.5% of middle-molecular toxins exceed 12,000 Da. - Removing these molecules are challenging since the clearance by diffusion decreases with the increase in the molecular weight of the solute. - Higher removal rate could be achieved using high-flux membranes by applying transmembrane pressure.	
Large molecules 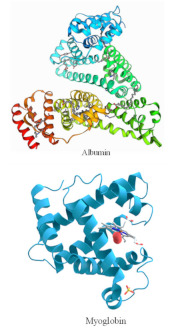	>15,000 Da	- Examples: myoglobin (17,000 Da), retinol-binding protein (21,000 Da) and albumin (66,700 Da) [16]. - Myoglobin should be mostly eliminated by convection due to its relatively high molecular weight [55]. - Large molecules, such as albumin, must be retained (at least 90%) after the dialysis process.	
Protein-bound toxins 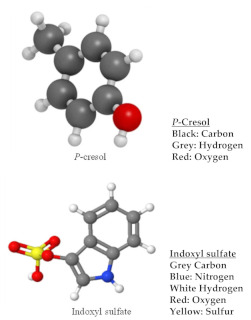	<500 Da	- Examples: indoxyl sulfate (213 Da), p-cresol (108 Da), indoxyl glucuronide (309 Da) and hippuric acid (179 Da) [56]. - A total of 27.8% of all uremic toxins are protein-bound toxins, and most of them have a molecular weight below 500 Da [57]. - Protein-bound toxins are strongly protein bound and are difficult to remove with conventional hemodialysis treatment [58]. - Some studies proposed a combination of hemodialysis and adsorption technology for effective removal of these toxins [16,58]. - P-cresol is a well-known protein-bound toxin because it affects many biological functions [59].	

**Table 4:** Ranges of dialysate compositions [48].

Composition of Dialysate	Concentration (mEq/L)
Sodium (Na)	135–145
Potassium (K)	0–4
Calcium (Ca)	2.5–3.5
Magnesium (Mg)	0.5–1.5
Bicarbonate (HCO_3_)	35–40
Acetate/Citrate	4–10/2.4
Glucose	0–200 mg/dl
Chloride (Cl)	98–112

**Table 5:** Advantages and disadvantages of different polymers used for hemodialysis membrane fabrication.

^a^ Polymer	Advantages	Disadvantages	Ref.
PSf and PES	- High thermal and mechanical stability - Good chemical and pH resistance	- Hydrophobicity - Oxidative stress	[69,70]
PAN	- Good blood compatibility - Reduced anaphylatoxin formed	- Negatively charged surface might activate dialyzer reactions	[5,71]
PA	- Wide pH tolerance - High thermal and mechanical stability	- Hydrophobicity - Low chemical resistance - Low β_2_-M sieving coefficient	[72]
CTA	- Good solute permeability - Symmetric membrane structure - Larger mean pore size	- Low biocompatibility	[57]
EVAL	- Larger mean pore size - Effective for high-molecular-weight toxins removal - Able to reduce oxidative stress and inflammation	- Loss of albumin	[73]

^a^ CTA (cellulose triacetate); EVAL (ethylene vinyl alcohol copolymer); PA (polyamide); PAN (polyacrylonitrile); PES (polyethersulfone); PSf (polysulfone).

**Table 6:** Summary of the performances of lab-synthesized membranes intended for hemodialysis applications.

Membranes	Pure Water Flux (Lm^−2^h^−1^bar^−1^)	Dialysis Performance			Other Membrane Features	Ref
		Urea Clearance (%)	Lysozyme Clearance (%)	BSA Retention (%)		
PES/SPES	182.6	-	-	99.9	- Fouling reduction. - Prolonged blood coagulation time. - Improved blood compatibility.	[77]
PLA/immobilized heparin	65	74.6	13.7	90.8	- Improved membrane blood compatibility, including platelet adhesion, prolongation of PRT time and decreased hemolysis rate.	[79]
PSf-EDA-26/PLA	54.00	67.5	22.5	95.0	- Improved mechanical and thermal stability.	[80]
PLA-PHEMA	236.7	70	50	69	- Improved hydrophilicity and antifouling property. - Better hemocompatibility.	[81]
PVA/PAN TFNC	290.0	82.6	45.8	95.0	- Excellent dialysis performances, especially for middle-molecule uremic toxins. - Better blood compatibility (platelet adhesion, blood coagulation time and BSA adsorption).	[82]
PES amphiphilic block copolymer	67.14	10.0	38.4	96.6	- Better blood compatibility (BSA adsorption, platelet adhesion and blood coagulation time). - Good antifouling property.	[93]
PES/comb-like amphiphilic block copolymer	96.07	-	-	94.2	- Improved water flux and protein antifouling properties. - -Improved hemocompatibility of the membranes.	[94]
PES/CA-g-PU	200.0	-	-	-	- Improved blood compatibility of the membrane (BSA and BFG adsorption, suppressed platelet adhesion and prolonged whole blood clotting time).	[95]
PES/MWCNT	68.5	56.0	28.0	90.0	- Improved uremic toxin removal, such as urea, creatinine and lysozyme.	[104]
PES/PCA-g-MWCNT	95.36	-	-	95.2	- Improved protein antifouling property.	[31]
PSf/E-TPGS	38.57	65.6	30.9	90.0	- Enhanced biocompatibility properties. - Better antioxidative property. - Improved solute rejection and urea clearance.	[111]
GO-doping PES	118.46	86.0	-	93.5	- Improved hemocompatibility and cell attachment proliferation.	[107]
PSf/Fe_2_O_3_	110.47	82.0	46.7	99.9	- Improved hemocompatibility of the membrane.	[109]
PES-TPGS-NZ	206.00	34.7	-	93.4	- Improved biocompatibility and improved attachment and proliferation of HEK-293 cells.	[112]

**Table 7:** Sterilization techniques and their conditions for dialyzers.

Sterilization Technique	Conditions	Ref.
Dry heat sterilization	- High sterilization temperature (up to 215 °C) - Moisture removal is needed when heat sterilization is conducted at a temperature of <100 °C	[123]
Ethylene oxide	- Compatible with many dialyzers - Low sterilization temperature (<60 °C) - Rapid activity, nontoxic and cost effective	[119,128]
Hydrogen peroxide	- Short cycle and low temperature (<60 °C) - No aeration requirement - No chemical residues	[129]
Steam sterilization	- High steam temperature at 121 °C for 20 min - Simple technique and rapid activity - Environmentally friendly - Low-cost sterilization	[121,130]
Gamma irradiation	- Compatible with many dialyzers - Promotes cross-linking between PSf and hydrophilizing agent	[122,127]

**Table 8:** Performance comparison of selected commercial PSf dialyzers on the market.

Dialyzer Brand		Asahi			Baxter			Fresenius			Toray		
Product Name		REXEED			Revaclear-400			FX80			Toraysulfone		
Blood Flow (mL/min)		200	300	400	200	300	400	200	300	400	200	300	400
^a^ Clearance in vitro (mL/min)	Urea	198	280	330	198	281	338	197	276	362	198	277	332
	Creatinine	194	265	309	191	267	315	189	250	287	196	264	308
	Phosphate	190	250	289	185	255	297	185	239	272	196	258	297
	Vitamin B_12_	152	183	197	158	191	213	148	175	190	162	202	226
	Inulin (Q_b_ = 200 mL/min)	92	-	-	84	-	-	112	125	133	131	162	182
K_o_A (mL/min)		1415			1439			1394			1035		
K_uf_ (mL/h.mmHg)		81			54			59			51		
Effective surface area (m^2^)		1.8			1.8			1.8			1.8		
Sieving coefficient	Inulin	1.0			1.0			1.0			0.9		
	β_2_-M	0.8			0.95			0.7			0.7		
	Myoglobin	0.4			0.68			0.1			0.2		
	Albumin	0.001			0.0027			<0.001			<0.001		

^a^ The in vitro performances were evaluated with blood flowrate varied in the range of 200–400 mL/min while dialysate flowrate was fixed at 500 mL/min. Testing temperature is 37 °C.

## Data Availability

Not applicable.
